# Supplementary material for: Cancer risk in individuals with intellectual disability in Sweden: A population-based cohort study
Source: PLoS Med. 2021 Oct 21;18(10):e1003840. doi: 10.1371/journal.pmed.1003840 (PMC8568154; doi:10.1371/journal.pmed.1003840)

S1 Fig. Hazard ratios (HRs) of cancer among individuals with intellectual disability (ID) by sex, compared to reference group

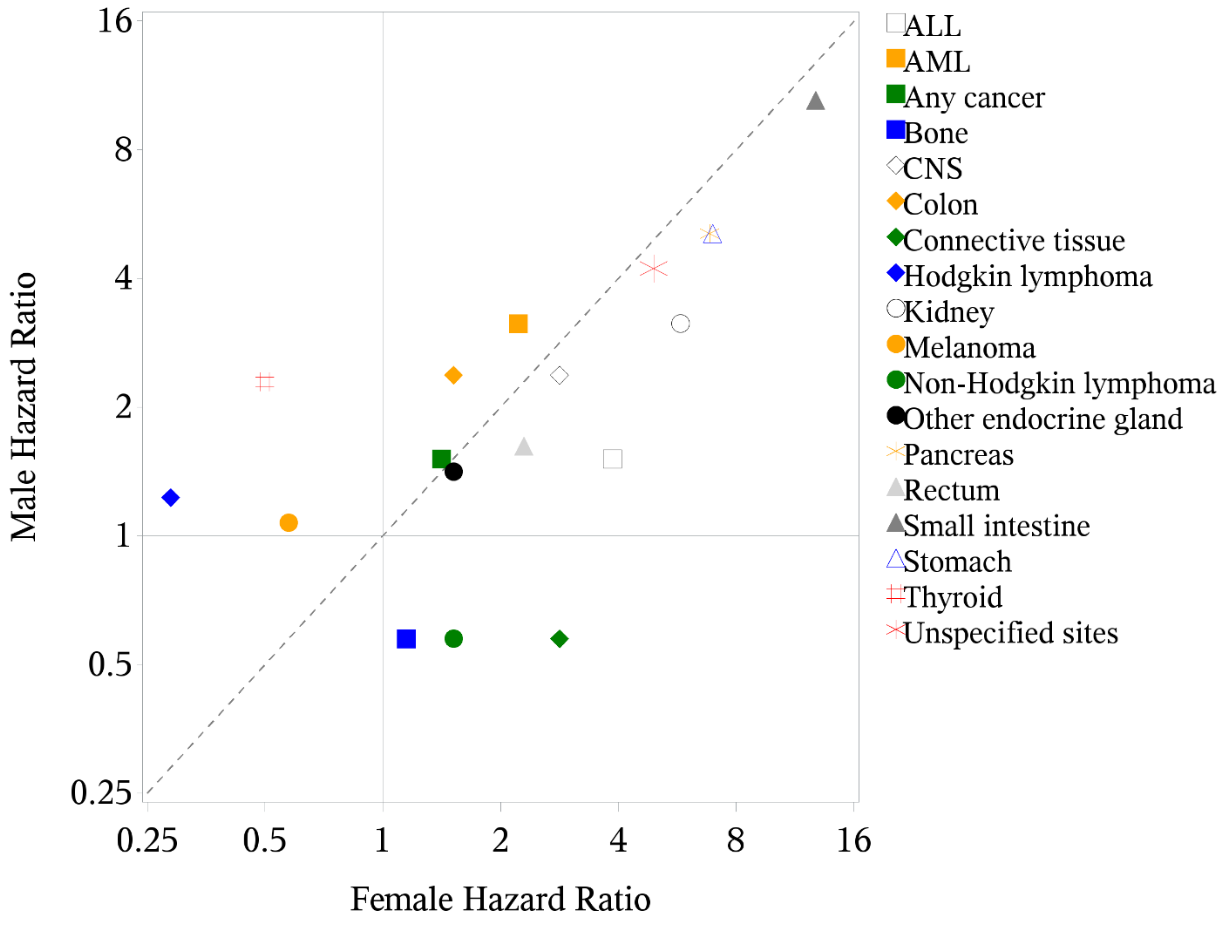

Supplement: S1 Fig — (PDF) [file pmed.1003840.s001.pdf]
