# Supplementary material for: Cancer risk in individuals with intellectual disability in Sweden: A population-based cohort study
Source: PLoS Med. 2021 Oct 21;18(10):e1003840. doi: 10.1371/journal.pmed.1003840 (PMC8568154; doi:10.1371/journal.pmed.1003840)

S3 Fig. Cancer risk overall (any cancer) and by cancer type among individuals with ID and reference group

A. Risk of any cancer.

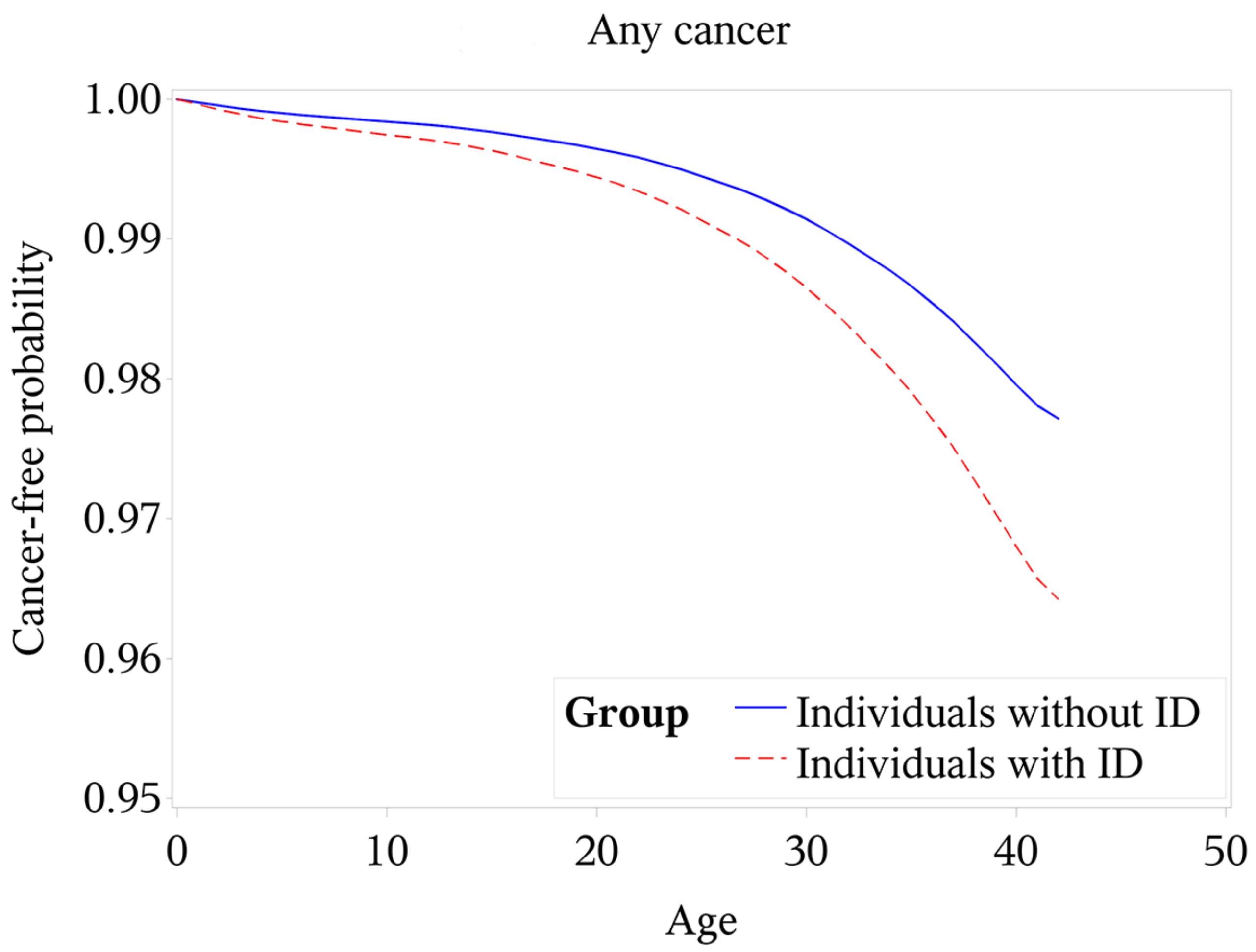

B. Risk of cancer of salivary gland, esophagus, stomach and small intestine.

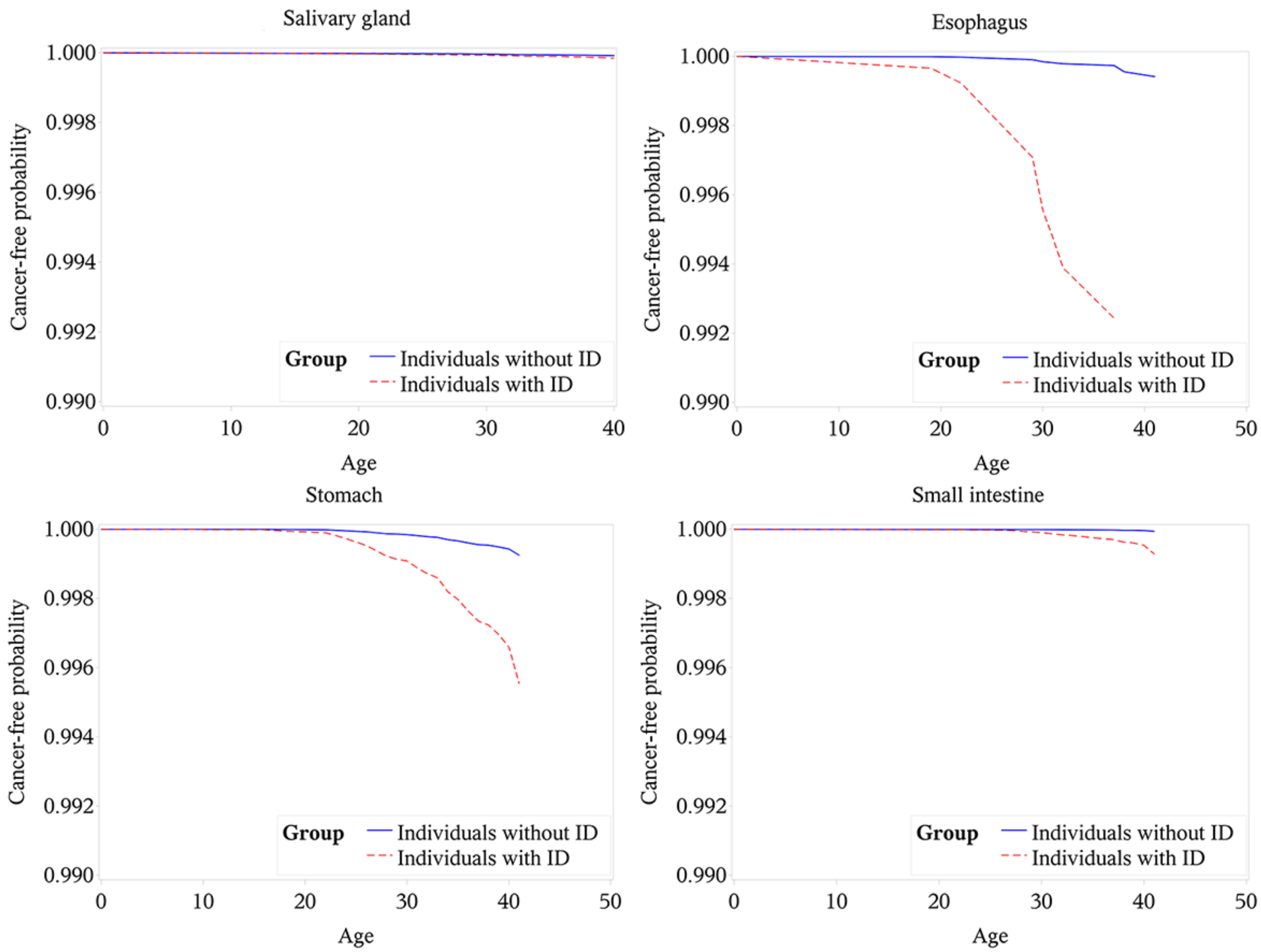

C. Risk of cancer of colon, rectum, liver and pancreas

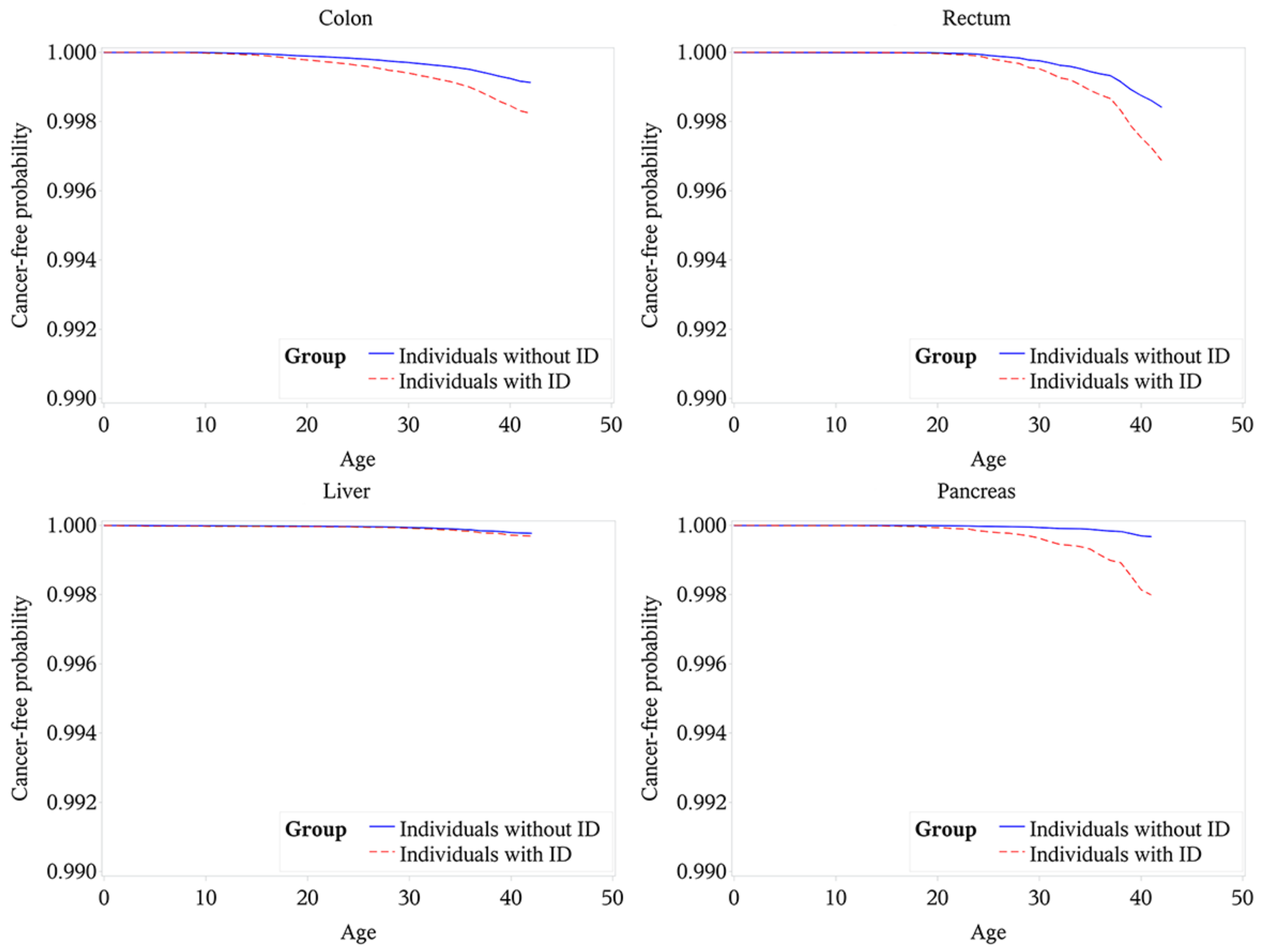

D. Risk of cancer of lung, breast, cervix and uterus

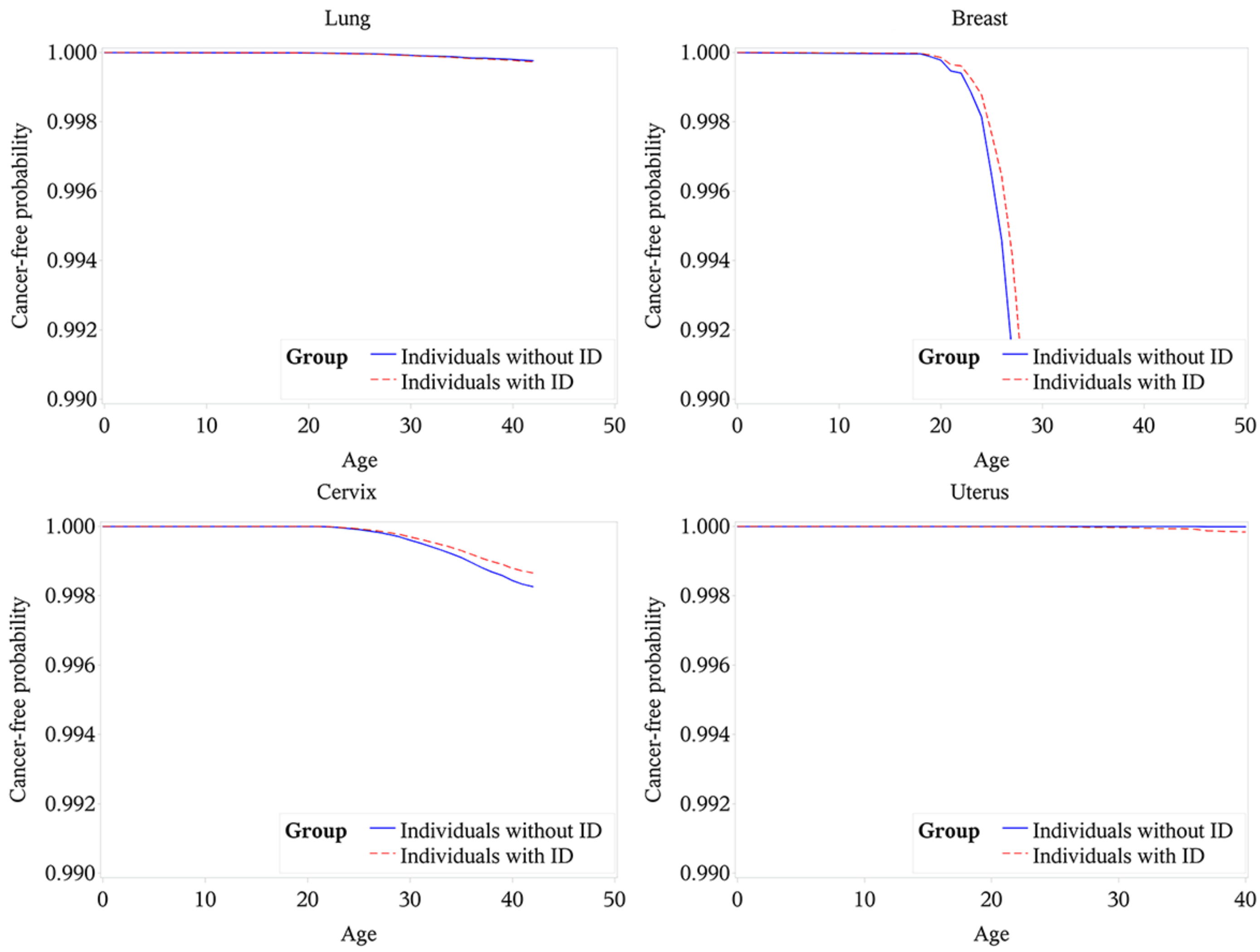

E. Risk of cancer of ovary, testis, kidney and melanoma

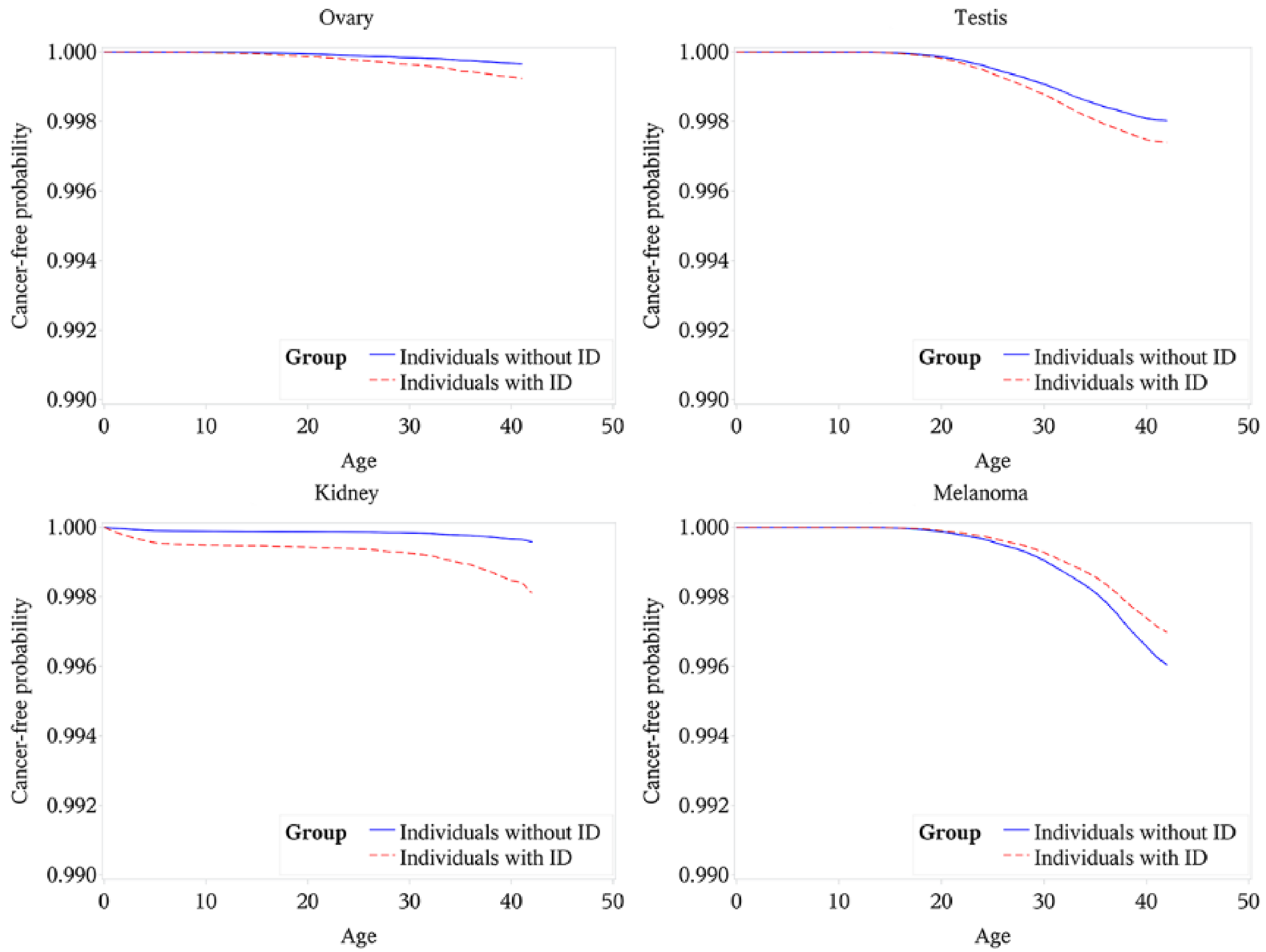

F. Risk of cancer of non-melanoma skin, eye, CNS, and thyroid

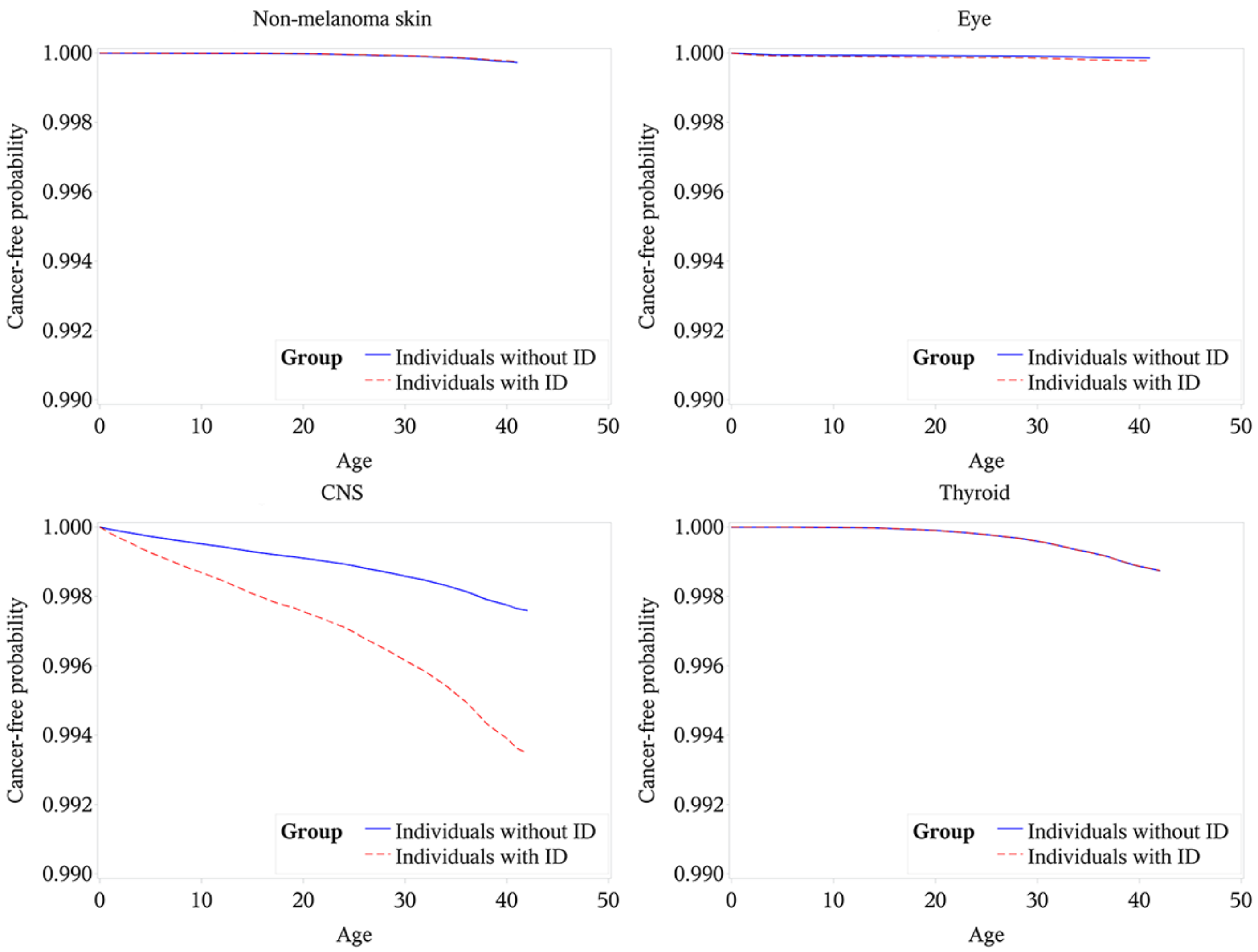

G. Risk of cancer of other endocrine gland, bone, connective tissue and other or unspecified sites

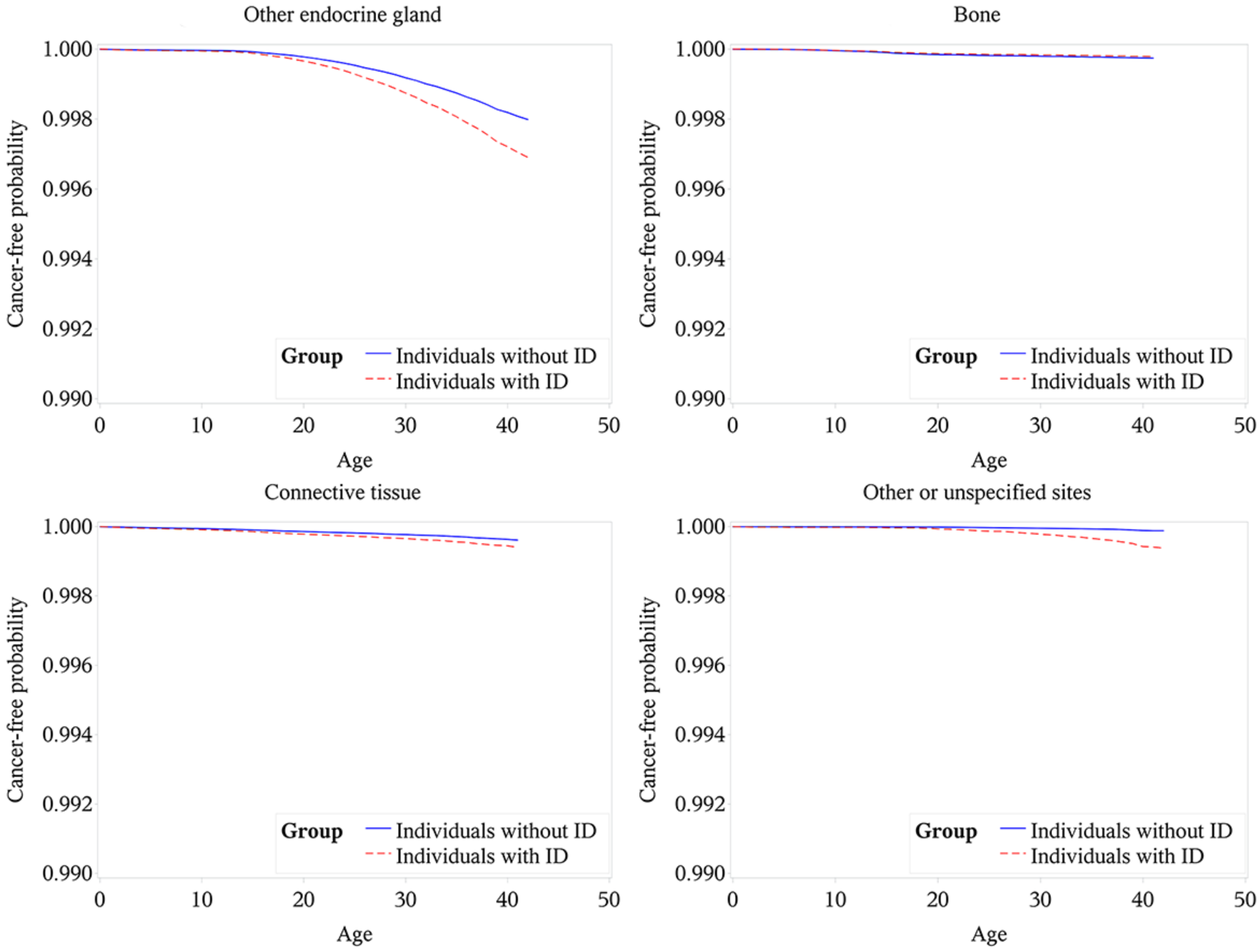

H. Risk of Hodgkin's lymphoma, Non-Hodgkin's lymphoma, ALL and AML

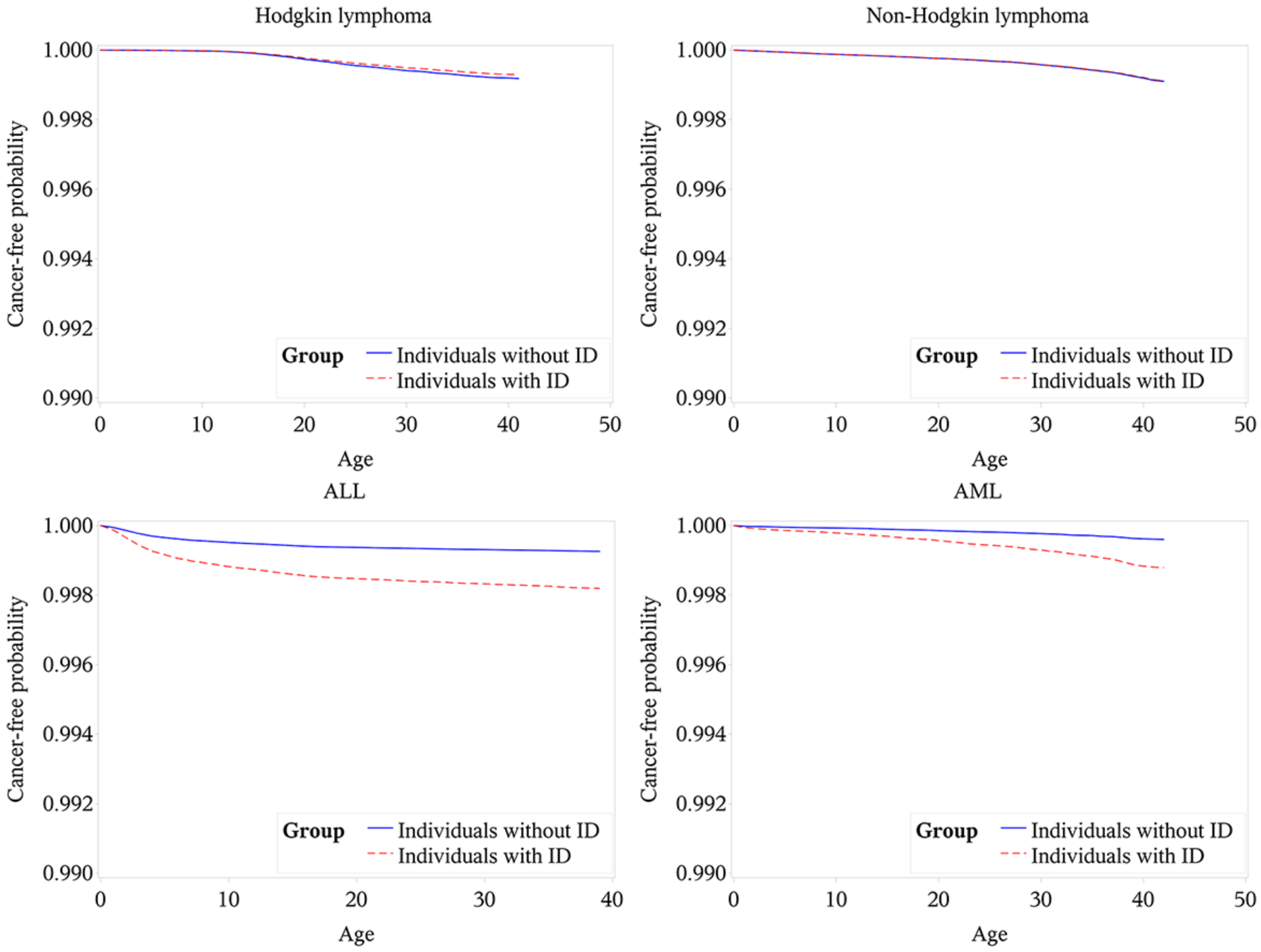

Supplement: S3 Fig — (PDF) [file pmed.1003840.s003.pdf]
