# Supplementary material for: Cancer risk in individuals with intellectual disability in Sweden: A population-based cohort study
Source: PLoS Med. 2021 Oct 21;18(10):e1003840. doi: 10.1371/journal.pmed.1003840 (PMC8568154; doi:10.1371/journal.pmed.1003840)
Supplement: S4 Fig — (PDF) [file pmed.1003840.s004.pdf]

S4 Fig. Hazard ratios (HRs) of cancer among individuals with intellectual disability (ID) by heritability[1] of cancer, compared to reference group

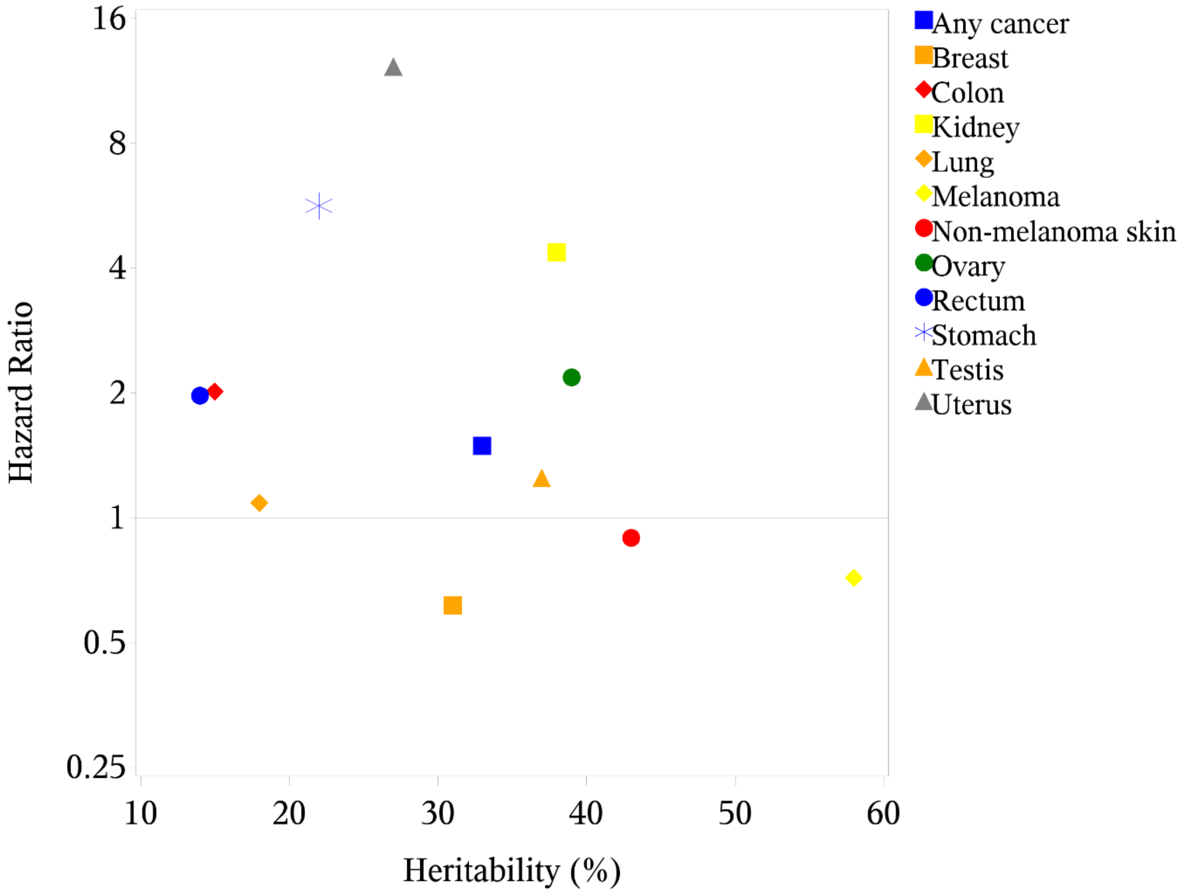

Reference

1. Mucci LA, Hjelmborg JB, Harris JR, Czene K, Havelick DJ, Scheike T, et al. Familial Risk and Heritability of Cancer Among Twins in Nordic Countries. JAMA. 2016;315(1):68-76. Epub 2016/01/10.
