# Supplementary material for: Cancer risk in individuals with intellectual disability in Sweden: A population-based cohort study
Source: PLoS Med. 2021 Oct 21;18(10):e1003840. doi: 10.1371/journal.pmed.1003840 (PMC8568154; doi:10.1371/journal.pmed.1003840)
Supplement: S1 Table — (PDF) [file pmed.1003840.s006.pdf]

S1 Table. ICD codes for intellectual disability (ID), severity of ID[a], ID type[b], intelligence quotient (IQ)[c], psychiatric disorders, and congenital malformations and chromosomal abnormalities

Variables	ICD codes
**ID**	ICD-8: 310-315<br><br>ICD-9: 317, 318, 318A, 318B, 318C (A, B, C are special Swedish codes), 319<br><br>ICD-10: F70-F73, F78, F79
**Severity of ID**	Mild ID (IQ: 50-69)[d]: ICD-8: 310, 311; ICD-9: 317; ICD-10: F70<br>Moderate ID (IQ: 35-49): ICD-8: 312; ICD-9: 318A; ICD-10: F71<br>Severe ID (IQ: 20-34): ICD-8: 313; ICD-9: 318B; ICD-10: F72<br>Profound ID (IQ: <20): ICD-8: 314; ICD-9: 318C; ICD-10: F73<br>Unspecified or other ID: ICD-8: 315; ICD-9: 319; ICD 10: F78-F79
**Psychiatric disorders**	ICD-8: 290-315<br><br>ICD-9: 290-319<br><br>ICD-10: F00-F99
**Congenital malformations and chromosomal abnormalities**	ICD-8: 310.4-310.5, 311.4-311.5, 312.4-312.5, 313.4-313.5, 314.4-314.5, 315.4-315.5, and 740-759<br><br>ICD-9: 740-759<br><br>ICD-10 Q00-Q99

[a] Borderline ID was included in mild ID.

[b] ID type includes two subgroups by mechanism: syndromic ID and idiopathic ID. Syndromic ID refers to individuals with both ICD codes of ID and ICD codes of congenital malformations and chromosomal abnormalities in the National Patient Register. Idiopathic ID refers to individuals with ICD codes of ID but without ICD codes of congenital malformations and chromosomal abnormalities in the National Patient Register.

[c] IQ score is part of ICD system to classify severity of ID. [World Health Organization. (1992). The ICD-10 classification of mental and behavioural disorders: clinical descriptions and diagnostic guidelines. World Health Organization. https://apps.who.int/iris/handle/10665/37958]

[d] In the analysis of association between IQ level and cancer risk, we first calculated the average IQ value for all individuals with ID (IQ_{all_avg}): $IQ_{all\_avg} = (\sum IQ_{mild} + \sum IQ_{moderate} + \sum IQ_{severe} + \sum IQ_{profound}) / N_{total} = (IQ_{mild\_avg} \times N_{mild} + IQ_{moderate\_avg} \times N_{moderate} + IQ_{severe\_avg} \times N_{severe} + IQ_{profound\_} \times N_{profound}) / N_{total}$ (N_{mild} represents number of individuals with mild ID, $N_{moderate}$ represents number of individuals with moderate ID, N_{severe} represents number of individuals with severe ID, $N_{profound}$ represents number of individuals with profound ID, and N_{total} represents total number of individuals with ID). Then we generated new

transformation of average IQ value for each subgroups, using average IQ value for each subgroup minus average value for all individuals with ID: $IQ_{mild\_trans} = IQ_{mild\_avg} - IQ_{all\_avg}$; $IQ_{moderate\_trans} = IQ_{moderate\_avg} - IQ_{all\_avg}$; $IQ_{severe\_trans} = IQ_{severe\_avg} - IQ_{all\_avg}$; $IQ_{profound\_trans} = IQ_{profound\_avg} - IQ_{all\_avg}$; ($IQ_{mild\_trans}$, $IQ_{moderate\_trans,}$ IQ_{severe_trans} and $IQ_{profound\_trans}$ represents transformed IQ value for individuals with mild ID, moderate ID, severe Id and profound ID, separately.
