# Supplementary material for: Cancer risk in individuals with intellectual disability in Sweden: A population-based cohort study
Source: PLoS Med. 2021 Oct 21;18(10):e1003840. doi: 10.1371/journal.pmed.1003840 (PMC8568154; doi:10.1371/journal.pmed.1003840)
Supplement: S2 Table — (PDF) [file pmed.1003840.s007.pdf]

S2 Table. Numbers of cancer cases among individuals with ID and individuals without ID.

ICD-7	Cancer types	Individuals without ID<br>N (%)[a]	Individuals with ID<br>N (%)[b]
140	Malignant neoplasm of lip	9 (0.04%)	
141	Malignant neoplasm of tongue	66 (0.26%)	
142	Malignant neoplasm of salivary gland	94 (0.38%)	1 (0.53%)
143	Malignant neoplasm of floor of mouth	5 (0.02%)	
144	Malignant neoplasm of other parts of mouth, and of mouth, unspecified	48 (0.19%)	
145	Malignant neoplasm of oral mesopharynx	16 (0.06%)	
146	Malignant neoplasm of nasopharynx	46 (0.18%)	
147	Malignant neoplasm of hypopharynx	2 (0.01%)	
148	Malignant neoplasm of pharynx, unspecified	2 (0.01%)	
150	Malignant neoplasm of oesophagus	9 (0.04%)	2 (1.06%)
151	Malignant neoplasm of stomach	57 (0.23%)	2 (1.06%)
152	Malignant neoplasm of small intestine, including duodenum	29 (0.12%)	2 (1.06%)
153	Malignant neoplasm of large intestine, except rectum	711 (2.85%)	11 (5.85%)
154	Malignant neoplasm of rectum	177 (0.71%)	2 (1.06%)
155	Malignant neoplasm of biliary passages and of liver (stated to be primary site)	206 (0.83%)	1 (0.53%)
156	Malignant neoplasm of liver (secondary and unspecified)	10 (0.04%)	
157	Malignant neoplasm of pancreas	50 (0.20%)	2 (1.06%)
158	Malignant neoplasm of peritoneum	11 (0.04%)	

ICD-7	Cancer types	Individuals without ID<br>N (%)[a]	Individuals with ID<br>N (%)[b]
160	Malignant neoplasm of nose, nasal cavities, middle ear and accessory sinuses	33 (0.13%)	
161	Malignant neoplasm of larynx	12 (0.05%)	
162	Malignant neoplasm of bronchus and trachea, and of lung specified as primary	159 (0.64%)	2 (1.06%)
163	Malignant neoplasm of lung, unspecified as to whether primary or secondary	3 (0.01%)	
164	Malignant neoplasm of mediastinum	31 (0.12%)	
170	Malignant neoplasm of breast	1,538 (6.16%)	5 (2.66%)
171	Malignant neoplasm of cervix uteri	1,224 (4.90%)	5 (2.66%)
172	Malignant neoplasm of Uterine uteri	55 (0.22%)	2 (1.06%)
173	Malignant neoplasm of other parts of uterus, including chorionepithelioma	22 (0.09%)	
174	Malignant neoplasm of uterus, unspecified	11 (0.04%)	1 (0.53%)
175	Malignant neoplasm of ovary, Fallopian tube and broad ligament	373 (1.49%)	4 (2.13%)
176	Malignant neoplasm of other and unspecified female genital organs	32 (0.13%)	
177	Malignant neoplasm of prostate	18 (0.07%)	
178	Malignant neoplasm of testis	2,263 (9.07%)	19 (10.1%)
179	Malignant neoplasm of other and unspecified male genital organs	23 (0.09%)	
180	Malignant neoplasm of kidney	628 (2.52%)	7 (3.72%)
181	Malignant neoplasm of bladder and other urinary organs	146 (0.58%)	
190	Malignant melanoma of skin	2,601 (10.4%)	11 (5.85%)

ICD-7	Cancer types	Individuals without ID N (%)[a]	Individuals with ID N (%)[b]
191	Other malignant neoplasm of skin	202 (0.81%)	1 (0.53%)
192	Malignant neoplasm of eye	402 (1.61%)	1 (0.53%)
193	Malignant neoplasm of brain and other parts of nervous system	4,131 (16.6%)	46 (24.5%)
194	Malignant neoplasm of thyroid gland	926 (3.71%)	6 (3.19%)
195	Malignant neoplasm of other endocrine glands	1,448 (5.80%)	12 (6.38%)
196	Malignant neoplasm of bone (including jaw bone)	538 (2.16%)	2 (1.06%)
197	Malignant neoplasm of connective tissue	667 (2.67%)	4 (2.13%)
199	Malignant neoplasm of other and unspecified sites	153 (0.61%)	4 (2.13%)
200	Malignant non-Hodgkin's lymphoma	955 (3.83%)	2 (1.06%)
201	Hodgkin's lymphoma	1,273 (5.10%)	6 (3.19%)
202	Other forms of lymphoma (reticulosis)	255 (1.02%)	2 (1.06%)
203	Multiple myeloma (plasmocytoma)	27 (0.11%)	
204	Lymphatic leukemia	2,192 (8.78%)	12 (6.38%)
205	Myeloid leukemia	737 (2.95%)	9 (4.79%)
206	Monocytic leukemia	56 (0.22%)	
207-209	Other hematological malignancies	278 (1.11%)	2 (1.06%)

[a] Percentage of all cancer cases among individuals without ID.

[b] Percentage of all cancer cases among individuals with ID.
