# Supplementary material for: Cancer risk in individuals with intellectual disability in Sweden: A population-based cohort study
Source: PLoS Med. 2021 Oct 21;18(10):e1003840. doi: 10.1371/journal.pmed.1003840 (PMC8568154; doi:10.1371/journal.pmed.1003840)
Supplement: S3 Table — (PDF) [file pmed.1003840.s008.pdf]

S3 Table. ICD codes for studied cancer types.

**Cancer types**	**ICD-7**
**Any cancer**	140-209
**By sites**	
Salivary gland	142
Esophagus	150
Stomach	151
Small intestine	152
Colon	153
Rectum	154
Liver	155
Pancreas	157
Lung	162
Breast	170
Cervix	171
Uterus	172-174
Ovary	175
Testis	178
Kidney	180
Melanoma	190
Non-melanoma skin	191
Eye	192
Central nervous system (CNS)	193
Thyroid	194
Other endocrine gland	195
Bone	196
Connective tissue	197
Other or unspecified sites	199
Hodgkin’s lymphoma	201
Non-Hodgkin’s lymphoma	200, 202, 2041
Acute lymphoid leukemia (ALL)	2040, 2049
Acute myeloid leukemia (AML)	2050, 2059, 2060, 2069
**By organ systems**	
Buccal cavity and pharynx	140-148
Digestive system	150-159

**Cancer types**	**ICD-7**
Respiratory system	160-165
Breast and reproductive system	170-179
Urinary system	180-181
Hematological malignancy	200-209
Other categories	190-199 (excluding 193)
