# Supplementary material for: Cancer risk in individuals with intellectual disability in Sweden: A population-based cohort study
Source: PLoS Med. 2021 Oct 21;18(10):e1003840. doi: 10.1371/journal.pmed.1003840 (PMC8568154; doi:10.1371/journal.pmed.1003840)
Supplement: S4 Table — (PDF) [file pmed.1003840.s009.pdf]

S4 Table. Birth characteristics, parental education and maternal smoking during pregnancy of the cohort participants

**Characteristics**	**Individuals without ID**<br>**(reference group)**<br>**Participants (%)**	**Individuals with ID**<br>**Participants (%)**	**ID-free full siblings of individuals with ID**<br>**Participants (%)**
**No. of individuals**	3,473,708	27,956	29,641
**Multiple birth**	84,431 (2.4%)	986 (3.5%)	1,114 (3.8%)
**Gestational age**			
<37 weeks	202,256 (5.8%)	3,897 (13.9%)	2,346 (7.9%)
37-41 weeks	2,990,528 (86.1%)	21,895 (78.3%)	25,259 (85.2%)
>41 weeks	280,924 (8.1%)	2,164 (7.7%)	2,036 (6.9%)
**Birth weight**			
<2.5 kg	146,001 (4.2%)	3,742 (13.4%)	1,687 (5.7%)
2.5-4 kg	2,695,168 (77.6%)	20,345 (72.8%)	22,643 (76.4%)
>4 kg	632,539 (18.2%)	3,869 (13.8%)	5,311 (17.9%)
**Apgar score at 1 minute**			
≤3	38,153 (1.1%)	1,151 (4.2%)	311 (1.1%)
4-6	108,571 (3.2%)	1,972 (7.2%)	973 (3.3%)
≥7	3,296,919 (95.7%)	24,284 (88.6%)	28,028 (95.6%)
Missing	30,065	549	329
**Maternal education at delivery**			
<9 years	4,863 (0.2%)	194 (1.1%)	167 (1.0%)
9-12 years	1,197,259 (59.6%)	13,564 (78.4%)	13,245 (75.4%)
>12 years	807,609 (40.2%)	3,537 (20.5%)	4,159 (23.7%)
Missing	1,463,977	10,661	12,070
**Paternal education at delivery**			
<9 years	20,080 (1.0%)	554 (3.2%)	530 (3.0%)
9-12 years	1,293,965 (64.9%)	13,454 (78.4%)	13,284 (75.9%)

**Characteristics**	**Individuals without ID**<br><br>**(reference group)**<br><br>**Participants (%)**	**Individuals with ID**<br><br>**Participants (%)**	**ID-free full siblings of individuals with ID**<br><br>**Participants (%)**
>12 years	678,338 (34.0%)	3,155 (18.4%)	3,690 (21.1%)
Missing	1,481,325	10,793	12,137
**Maternal smoking during pregnancy**			
Yes	410,246 (16.0%)	6,103 (27.7%)	5,612 (23.6%)
No	2,157,348 (84.0%)	15,969 (72.3%)	18,148 (76.4%)
Missing	906,114	5,884	5,881
