# Supplementary material for: Cancer risk in individuals with intellectual disability in Sweden: A population-based cohort study
Source: PLoS Med. 2021 Oct 21;18(10):e1003840. doi: 10.1371/journal.pmed.1003840 (PMC8568154; doi:10.1371/journal.pmed.1003840)
Supplement: S5 Table — (PDF) [file pmed.1003840.s010.pdf]

S5 Table. Characteristics of individuals with intellectual disability (ID) by severity of ID[a]

Characteristics	Mild ID<br>Individuals (%)	Moderate ID<br>Individuals (%)	Severe ID<br>Individuals (%)	Profound ID<br>Individuals (%)	Unspecified or other ID<br>Individuals (%)
**No. of individuals**	15,334	2,683	1,078	450	8,411
**Sex**					
Male	8,824 (57.6%)	1,642 (61.2%)	616 (57.1%)	236 (52.4%)	4,903 (58.3%)
Female	6,510 (42.4%)	1,041 (38.8%)	462 (42.9%)	214 (47.6%)	3,508 (41.7%)
**Age at diagnosis, years**					
≤5	1,266 (8.3%)	469 (17.5%)	382 (35.4%)	174 (38.7%)	4,210 (50.1%)
6-10	3,506 (22.9%)	755 (28.1%)	276 (25.6%)	95 (21.1%)	1,562 (18.6%)
11-15	4,387 (28.6%)	630 (23.5%)	210 (19.5%)	88 (19.6%)	1,021 (12.1%)
16-20	3,286 (21.4%)	495 (18.5%)	130 (12.1%)	41 (9.1%)	753 (9.0%)
>20	2,889 (18.8%)	334 (12.4%)	80 (7.4%)	52 (11.6%)	865 (10.3%)
**Birth year**					

Characteristics	Mild ID<br>Individuals (%)	Moderate ID<br>Individuals (%)	Severe ID<br>Individuals (%)	Profound ID<br>Individuals (%)	Unspecified or other ID<br>Individuals (%)
1974-1983	2,176 (14.2%)	444 (16.5%)	229 (21.2%)	100 (22.2%)	2,362 (28.1%)
1984-1993	6,188 (40.4%)	1,128 (42.0%)	484 (44.9%)	179 (39.8%)	2,347 (27.9%)
1994-2003	5,458 (35.6%)	785 (29.3%)	249 (23.0%)	122 (27.1%)	2,243 (26.7%)
2004-2013	1,512 (9.9%)	326 (12.2%)	116 (10.7%)	49 (10.9%)	1,459 (17.3%)
**Maternal education at delivery**					
>12 years	1,573 (15.6%)	402 (24.4%)	179 (29.4%)	78 (31.8%)	1,305 (27.9%)
9-12 years	8,413 (83.1%)	1,237 (74.9%)	424 (69.6%)	165 (67.4%)	3,325 (71.2%)
<9 years	133 (1.3%)	12 (0.7%)	6 (1.0%)	2 (0.8%)	41 (0.9%)
Missing	5,215	1,032	469	205	3,740
**Paternal education at delivery**					
>12 years	1,419 (14.1%)	358 (21.8%)	165 (27.4%)	67 (27.6%)	1,146 (24.7%)

Characteristics	Mild ID<br>Individuals (%)	Moderate ID<br>Individuals (%)	Severe ID<br>Individuals (%)	Profound ID<br>Individuals (%)	Unspecified or other ID<br>Individuals (%)
9-12 years	8,277 (82.4%)	1,234 (75.2%)	423 (70.1%)	167 (68.7%)	3,353 (72.4%)
<9 years	346 (3.5%)	49 (3.0%)	15 (2.5%)	9 (3.7%)	135 (2.9%)
Missing	5,292	1,042	475	207	3,777
**Maternal age at delivery, years**					
<20	571 (3.7%)	61 (2.3%)	22 (2.0%)	9 (2.0%)	227 (2.7%)
20-29	8,377 (54.7%)	1,332 (49.6%)	546 (50.6%)	228 (50.7%)	4,151 (49.4%)
30-39	5,906 (38.5%)	1,175 (43.8%)	462 (42.9%)	193 (42.9%)	3,712 (44.1%)
≥40	480 (3.1%)	115 (4.3%)	48 (4.5%)	20 (4.4%)	321 (3.8%)
**Paternal age at delivery, years**					
<20	136 (0.9%)	11 (0.4%)	4 (0.4%)	2 (0.4%)	49 (0.6%)
20-29	6,158 (40.2%)	947 (35.3%)	361 (33.5%)	170 (37.8%)	2,898 (34.5%)
30-39	7,113 (46.4%)	1,362 (50.8%)	557 (51.7%)	219 (48.7%)	4,371 (52.0%)
≥40	1,927 (12.6%)	363 (13.5%)	156 (14.5%)	59 (13.1%)	1,093 (13.0%)

Characteristics	Mild ID<br>Individuals (%)	Moderate ID<br>Individuals (%)	Severe ID<br>Individuals (%)	Profound ID<br>Individuals (%)	Unspecified or other ID<br>Individuals (%)
**Gestational age**					
<37 weeks	1,926 (12.6%)	389 (14.5%)	159 (14.8%)	91 (20.2%)	1,332 (15.8%)
37-41 weeks	12,283 (80.1%)	2,071 (77.2%)	830 (77.0%)	321 (71.3%)	6,390 (76.0%)
>41 weeks	1,125 (7.3%)	223 (8.3%)	89 (8.3%)	38 (8.4%)	689 (8.2%)
**Birth weight**					
<2.5 kg	1,738 (11.3%)	386 (14.4%)	208 (19.3%)	91 (20.2%)	1,319 (15.7%)
2.5-4 kg	11,366 (74.1%)	1,922 (71.6%)	741 (68.7%)	298 (66.2%)	6,018 (71.6%)
> 4 kg	2,230 (14.5%)	375 (14.0%)	129 (12.0%)	61 (13.6%)	1,074 (12.8%)
**Apgar score at 1 minute**					
≥7	13,790 (91.3%)	2,316 (88.3%)	819 (79.3%)	330 (76.4%)	7,029 (85.5%)
4-6	880 (5.8%)	181 (6.9%)	116 (11.2%)	51 (11.8%)	744 (9.1%)
≤3	431 (2.9%)	125 (4.8%)	98 (9.5%)	51 (11.8%)	446 (5.4%)
Missing	233	61	45	18	192
**Maternal smoking during pregnancy**					

Characteristics	Mild ID<br>Individuals (%)	Moderate ID<br>Individuals (%)	Severe ID<br>Individuals (%)	Profound ID<br>Individuals (%)	Unspecified or other ID<br>Individuals (%)
Yes	3,877 (30.3%)	519 (23.9%)	211 (25.1%)	87 (25.9%)	1,409 (23.7%)
No	8,905 (69.7%)	1,651 (76.1%)	630 (74.9%)	249 (74.1%)	4,534 (76.3%)
Missing	2,552	513	237	114	2,468
**Multiple birth**	522 (3.4%)	89 (3.3%)	31 (2.9%)	18 (4.0%)	326 (3.9%)
**Maternal history of psychiatric disorders at delivery**	1,891 (12.3%)	249 (9.3%)	79 (7.3%)	28 (6.2%)	847 (10.1%)
**Paternal history of psychiatric disorders at delivery**	1,629 (10.6%)	186 (6.9%)	61 (5.7%)	29 (6.4%)	721 (8.6%)
**Maternal history of cancer at delivery**	61 (0.4%)	6 (0.2%)	4 (0.4%)	1 (0.2%)	25 (0.3%)
**Paternal history of cancer at delivery**	48 (0.3%)	11 (0.4%)	2 (0.2%)	2 (0.4%)	41 (0.5%)

[a] Among all 27,956 individuals, 8,798 (31.5%) had varying status of ID severity during follow-up. For patients with varying status of ID severity, if patients had unspecified or other ID at first diagnosis and then had a specified diagnosis of ID (mild, moderate, severe, or profound ID) during follow-up, these patients were considered as specified ID (mild, moderate, severe, or profound ID).
