# Supplementary material for: Cancer risk in individuals with intellectual disability in Sweden: A population-based cohort study
Source: PLoS Med. 2021 Oct 21;18(10):e1003840. doi: 10.1371/journal.pmed.1003840 (PMC8568154; doi:10.1371/journal.pmed.1003840)
Supplement: S6 Table — (PDF) [file pmed.1003840.s011.pdf]

S6 Table. Association between IQ score and risk of cancer among individuals with intellectual disability, compared to reference group, by cancer type

Cancer type	HR[a]	P value
**Any cancer**	0.92	0.27
**Salivary gland**	1.05	0.97
**Esophagus**	0.54	0.13
**Stomach**	1.05	0.96
**Small intestine**	2.62	0.61
**Colon**	0.67	0.08
**Rectum**	1.06	0.95
**Liver**	1.09	0.94
**Pancreas**	2.04	0.65
**Lung**	Not estimable[e]	0.97
**Breast**	3.08	0.40
**Cervix**	9.34	0.26
**Uterus**	0.97	0.98
**Ovary**	0.87	0.77
**Testis**	0.97	0.91
**Kidney**	0.84	0.63
**Melanoma**	0.77	0.29
**Non-melanoma skin**	0.00	0.97
**Eye**	Not estimable[e]	0.97
**CNS[b]**	0.92	0.58
**Thyroid**	2.69	0.41
**Other endocrine gland**	0.94	0.82
**Bone**	2.18	0.63
**Connective tissue**	1.04	0.94
**Other or unspecified sites**	1.06	0.92
**Hodgkin's lymphoma**	0.73	0.28
**Non-Hodgkin's lymphoma**	1.06	0.91
**ALL[c]**	2.53	0.21
**AML[d]**	1.06	0.89

[a] Analyses adjusted for birth year (as natural cubic spline), sex, IQ score, maternal and paternal age at delivery, maternal and paternal psychiatric disorder history at delivery, maternal and paternal cancer history at delivery. HR refers to hazard ratio with increase of every 10 units of IQ score.

[b] CNS refers to central nervous system.

[c] ALL refers to acute lymphoid leukemia.

[d] AML refers to acute myeloid leukemia.

[e] Not estimable indicates lack of convergence due to lack of cancer cases.
