# Supplementary material for: Cancer risk in individuals with intellectual disability in Sweden: A population-based cohort study
Source: PLoS Med. 2021 Oct 21;18(10):e1003840. doi: 10.1371/journal.pmed.1003840 (PMC8568154; doi:10.1371/journal.pmed.1003840)
Supplement: S7 Table — (PDF) [file pmed.1003840.s012.pdf]

S7 Table. Hazard ratios (HRs) with 95% confidence intervals (CIs) of any cancer among individuals with intellectual disability (ID), compared to reference group, further separately adjusted for or stratified by maternal smoking during pregnancy, parental education at delivery, multiple birth, gestational age, birth weight, and Apgar score at 1 minute[a]

Models	HR (95% CI)
**Model 2[b]**	1.57 (1.35-1.82)
**Model 2+ maternal smoking**	1.57 (1.35-1.82)
**Model 2 (among individuals without maternal smoking)**	1.73 (1.44-2.07)
**Model 2 (among individuals with maternal smoking)**	1.13 (0.72-1.77)
**Model 2 + maternal education+ paternal education**	1.57 (1.35-1.82)
**Model 2 (among individuals with maternal education <9 years)**	-
**Model 2 (among individuals with 9 years ≤ maternal education ≤ 12 years)**	1.51 (1.22-1.88)
**Model 2 (among individuals with maternal education >12 years)**	1.74 (1.21-2.48)
**Model 2 (among individuals with paternal education <9 years)**	2.41 (0.82-7.05)
**Model 2 (among individuals with 9 years ≤ paternal education ≤ 12 years)**	1.58 (1.31-1.90)
**Model 2 (among individuals with paternal education >12 years)**	1.47 (1.01-2.15)
**Model 2 + multiple birth**	1.57 (1.35-1.82)
**Model 2 (among multiple births)**	1.94 (0.86-4.35)
**Model 2 (among singletons)**	1.56 (1.34-1.82)
**Model 2 + gestational age + birth weight + Apgar score at 1 minute**	1.56 (1.34-1.81)
**Model 2 (among individuals with gestational age <37 weeks)**	1.44 (0.94-2.19)
**Model 2 (among individuals with 37 weeks ≤ gestational age ≤41 weeks)**	1.60 (1.35-1.89)
**Model 2 (among individuals with 41 weeks < gestational age)**	1.46 (0.88-2.42)
**Model 2 (among individuals with Apgar score at 1 minute ≤3)**	0.40 (0.10-1.62)
**Model 2 (among individuals with 4 ≤ Apgar score at 1 minute ≤6)**	1.23 (0.68-2.24)
**Model 2 (among individuals with Apgar score at 1 minute ≥7)**	1.64 (1.41-1.92)
**Model 2 (among individuals with birth weight <2.5kg)**	1.64 (1.10-2.44)
**Model 2 (among individuals with 2.5kg ≤ birth weight ≤4kg)**	1.46 (1.22-1.75)
**Model 2 (among individuals with 4kg < birth weight)**	2.10 (1.47-3.01)

[a] Multivariable analysis was conducted with multiple imputation for missing values with Apgar score at 1 minute, maternal smoking during pregnancy, maternal education and paternal education by means of chained equations (5 imputations). The estimates from different imputed data sets were combined with the use of the Rubin's rules.

[b] Analyses adjusted for birth year (as natural cubic splines), sex, maternal and paternal age at delivery, maternal and paternal psychiatric disorder history at delivery, maternal and paternal cancer history at delivery.
