# Supplementary material for: Cancer risk in individuals with intellectual disability in Sweden: A population-based cohort study
Source: PLoS Med. 2021 Oct 21;18(10):e1003840. doi: 10.1371/journal.pmed.1003840 (PMC8568154; doi:10.1371/journal.pmed.1003840)
Supplement: S8 Table — (PDF) [file pmed.1003840.s013.pdf]

S8 Table. Subgroup analyses of pre-term birth: Incidence rates (IRs, per 100,000 person-years) and hazard ratios (HRs) with 95% confidence intervals (CIs) of cancer among individuals with intellectual disability (ID), compared to reference group, restricting analyses among individuals that were born pre-term

**Cancer types**	**IR among reference group**	**IR among individuals with ID**	**Model 2[a]**<br><br>**HR (95% CI)**
**Any cancer**	32.69	52.82	1.44 (0.94-2.19)
**Salivary gland**	0.16	-	-
**Esophagus**	-	2.39	-
**Stomach**	0.07	-	-
**Small intestine**	0.02	2.39	89.7 (5.6-1439.8)
**Colon**	0.82	-	-
**Rectum**	0.24	-	-
**Liver**	0.44	-	-
**Pancreas**	-	-	-
**Lung**	0.22	-	-
**Breast**	1.64	4.79	2.3 (0.6-9.6)
**Cervix**	1.35	2.39	1.2 (0.2-8.8)
**Uterus**	-	2.39	-
**Ovary**	0.42	4.79	10.0 (2.3-43.6)
**Testis**	3.35	11.97	2.3 (0.9-5.6)
**Kidney**	1.02	2.39	4.6 (0.6-34.6)
**Melanoma**	2.97	2.39	0.6 (0.1-4.1)
**Non-melanoma skin**	0.31	-	-
**Eye**	0.60	-	-
**CNS[b]**	5.82	7.18	1.3 (0.4-4.1)
**Thyroid**	1.00	2.39	1.6 (0.2-11.4)
**Other endocrine gland**	1.93	2.39	0.9 (0.1-6.5)
**Bone**	0.67	2.39	3.3 (0.4-24.3)
**Connective tissue**	1.09	-	-
**Other or unspecified sites**	0.22	-	-
**Hodgkin's lymphoma**	1.47	-	-

Cancer types	IR among reference group	IR among individuals with ID	Model 2[a]<br><br>HR (95% CI)
**Non-Hodgkin's lymphoma**	1.51	-	-
**ALL[c]**	2.89	-	-
**AML[d]**	0.95	-	-

[a] Analyses adjusted for birth year (as natural cubic splines), sex, maternal and paternal age at delivery, maternal and paternal psychiatric disorder history at delivery, maternal and paternal cancer history at delivery.

[b] CNS refers to central nervous system.

[c] ALL refers to acute lymphoid leukemia.

[d] AML refers to acute myeloid leukemia.

"-" refers to no cancer case.
