# Supplementary material for: Cancer risk in individuals with intellectual disability in Sweden: A population-based cohort study
Source: PLoS Med. 2021 Oct 21;18(10):e1003840. doi: 10.1371/journal.pmed.1003840 (PMC8568154; doi:10.1371/journal.pmed.1003840)
Supplement: S9 Table — (PDF) [file pmed.1003840.s014.pdf]

S9 Table. Incidence rates (IRs, per 100,000 person-years) and hazard ratios (HRs) with 95% confidence intervals (CIs) of childhood cancer (age ≤ 18 years) among individuals with intellectual disability (ID) by cancer type, compared to reference group[a]

Cancer types	IR among reference group	IR among individuals with ID	Model 1[b] HR (95% CI)	Model 2[c] HR (95% CI)
**Any cancer**	17.20	49.00	2.82 (2.19-3.62)	2.81 (2.18-3.62)
**Salivary gland**	0.06	0.71	8.1 (1.1-59.5)	8.3 (1.1-61.8)
**Esophagus**	-	-	-	-
**Stomach**	-	-	-	-
**Small intestine**	-	-	-	-
**Colon**	0.29	1.42	1.6 (0.2-11.1)	1.6 (0.2-11.1)
**Rectum**	0.01	-	-	-
**Liver**	0.20	-	-	-
**Pancreas**	0.01	-	-	-
**Lung**	0.03	-	-	-
**Breast**	-	-	-	-
**Cervix**	0.01	-	-	-
**Uterus**	-	-	-	-
**Ovary**	0.17	0.71	3.3 (0.5-23.5)	3.1 (0.4-22.0)
**Testis**	0.29	1.42	3.0 (0.7-12.1)	3.0 (0.7-12.2)
**Kidney**	0.91	3.55	9.5 (3.5-25.5)	9.2 (3.4-24.8)
**Melanoma**	0.19	-	-	-
**Non-melanoma skin**	0.06	-	-	-
**Eye**	0.65	0.71	3.6 (0.5-25.7)	3.5 (0.5-25.2)
**CNS[d]**	4.96	20.58	4.0 (2.7-6.0)	4.0 (2.7-6.0)
**Thyroid**	0.26	1.42	3.5 (0.9-14.2)	3.5 (0.9-14.4)
**Other endocrine gland**	0.65	1.42	1.7 (0.4-6.9)	1.8 (0.4-7.1)
**Bone**	0.71	0.71	0.7 (0.1-5.1)	0.7 (0.1-5.1)
**Connective tissue**	0.74	2.13	3.1 (1.0-9.7)	3.0 (1.0-9.5)
**Other or unspecified sites**	0.05	0.71	20.1 (2.6-155.0)	17.8 (2.3-138.6)
**Hodgkin's lymphoma**	0.73	3.54	2.9 (1.2-7.1)	3.0 (1.2-7.2)

Cancer types	IR among reference group	IR among individuals with ID	Model 1[b]<br><br>HR (95% CI)	Model 2[c]<br><br>HR (95% CI)
**Non-Hodgkin's lymphoma**	1.24	0.71	0.6 (0.1-4.3)	0.6 (0.1-4.3)
**ALL[e]**	3.80	4.96	1.7 (0.8-3.9)	1.7 (0.8-3.9)
**AML[f]**	0.76	2.84	4.3 (1.6-11.7)	4.4 (1.6-11.7)

[a] The analyses were conducted by restricting outcome as childhood cancer—cancer diagnosis before the age of 18.

[b] Analyses adjusted for birth year (as natural cubic spline) and sex.

[c] Analyses additionally adjusted for maternal and paternal age at delivery, maternal and paternal psychiatric disorder history at delivery, maternal and paternal cancer history at delivery.

[d] CNS refers to central nervous system.

[e] ALL refers to acute lymphoid leukemia.

[f] AML refers to acute myeloid leukemia.

"-" refers to no cancer case.
