# Supplementary material for: Cancer risk in individuals with intellectual disability in Sweden: A population-based cohort study
Source: PLoS Med. 2021 Oct 21;18(10):e1003840. doi: 10.1371/journal.pmed.1003840 (PMC8568154; doi:10.1371/journal.pmed.1003840)
Supplement: S10 Table — (PDF) [file pmed.1003840.s015.pdf]

S10 Table. Comparison between different calendar years: Incidence rates (IRs, per 100,000 person-years) and hazard ratios (HRs) with 95% confidence intervals (CIs) of cancer among individuals with intellectual disability (ID), compared to reference group, comparison between individuals born during 1994-2013 and those born during 1974-1993.

	**Individuals born during 1994-2013 and followed until 31 Dec 2016**			**Individuals born during 1974-1993 and followed until 31 Dec 1996**		
**Cancer types**	**IR among reference group**	**IR among individuals with ID**	**Model 2[a]**<br>**HR (95% CI)**	**IR among reference group**	**IR among individuals with ID**	**Model 2[a]**<br>**HR (95% CI)**
**Any cancer**	18.12	44.31	2.74 (1.92-3.91)	17.07	55.07	2.90 (1.75-4.82)
**Salivary gland**	0.06	1.34	14.3 (1.8-112.1)	0.03	-	-
**Esophagus**	-	-	-	-	-	-
**Stomach**	0.01	-	-	-	-	-
**Small intestine**	0.01	-	-	-	-	-
**Colon**	0.25	1.34	2.8 (0.4-20.1)	0.24	-	-
**Rectum**	0.01	-	-	0.01	-	-
**Liver**	0.29	-	-	0.20	-	-
**Pancreas**	0.01	-	-	-	-	-
**Lung**	0.05	-	-	0.02	-	-
**Breast**	-	-	-	-	-	-
**Cervix**	0.01	-	-	0.01	-	-
**Uterus**	-	-	-	-	-	-
**Ovary**	0.13	1.34	7.7 (1.0-57.8)	0.14	-	-
**Testis**	0.29	1.34	2.0 (0.3-14.9)	0.27	-	-

	Individuals born during 1994-2013 and followed until 31 Dec 2016			Individuals born during 1974-1993 and followed until 31 Dec 1996		
**Cancer types**	**IR among reference group**	**IR among individuals with ID**	**Model 2[a]**<br><br>**HR (95% CI)**	**IR among reference group**	**IR among individuals with ID**	**Model 2[a]**<br><br>**HR (95% CI)**
**Kidney**	1.10	4.02	10.0 (2.5-40.8)	1.05	5.79	12.7 (3.1-51.4)
**Melanoma**	0.20	-	-	0.18	-	-
**Non-melanoma skin**	0.07	-	-	0.05	-	-
**Eye**	0.83	1.34	6.8 (0.9-49.8)	0.69	-	-
**CNS[b]**	4.93	16.09	4.1 (2.2-7.4)	5.08	28.97	4.5 (2.1-9.5)
**Thyroid**	0.29	1.34	2.6 (0.4-19.1)	0.17	-	-
**Other endocrine gland**	0.79	1.34	1.4 (0.2-9.9)	0.42	2.89	8.6 (1.2-62.6)
**Bone**	0.59	-	-	0.62	-	-
**Connective tissue**	0.79	2.68	3.3 (0.8-13.6)	0.78	2.89	4.2 (0.6-30.4)
**Other or unspecified sites**	0.05	-	-	0.06	2.89	89.2 (10.6-750.2)
**Hodgkin's lymphoma**	0.58	2.68	2.2 (0.5-8.8)	0.65	-	-
**Non-Hodgkin's lymphoma**	1.31	1.34	1.1 (0.2-8.1)	1.19	-	-
**ALL[c]**	4.13	5.36	2.5 (0.9-6.8)	4.16	5.79	0.9 (0.1-6.5)
**AML[d]**	0.92	2.68	4.2 (1.0-17.3)	0.63	2.89	6.2 (0.9-44.8)

[a] Analyses adjusted for birth year (as natural cubic splines), sex, maternal and paternal age at delivery, maternal and paternal psychiatric disorder history at delivery, maternal and paternal cancer

history at delivery.

[b] CNS refers to central nervous system.

[c] ALL refers to acute lymphoid leukemia.

[d] AML refers to acute myeloid leukemia.

“-“ refers to no cancer case.
