# Supplementary material for: Cancer risk in individuals with intellectual disability in Sweden: A population-based cohort study
Source: PLoS Med. 2021 Oct 21;18(10):e1003840. doi: 10.1371/journal.pmed.1003840 (PMC8568154; doi:10.1371/journal.pmed.1003840)
Supplement: S11 Table — (PDF) [file pmed.1003840.s016.pdf]

S11 Table. Incidence rates (IRs, per 100,000 person-years) and hazard ratios (HRs) with 95% confidence intervals (CIs) of cancer among individuals with intellectual disability (ID) by organ systems, compared to reference group

**Cancer categories**	**Model 2[a]**
Buccal cavity and pharynx	0.6 (0.1-4.4)
Digestive system	2.9 (1.8-4.6)
Respiratory system	0.8 (0.1-5.4)
Breast and reproductive system	1.2 (0.8-1.6)
Urinary system	2.8 (1.3-6.3)
CNS[b]	2.7 (2.0-3.7)
Hematological malignancy	1.5 (1.1-2.2)
Other categories	1.2 (0.9-1.6)

[a] Analyses adjusted for birth year (as natural cubic splines), sex, maternal and paternal age at delivery, maternal and paternal psychiatric disorder history at delivery, maternal and paternal cancer history at delivery.

[b] CNS refers to central nervous system.
