# Supplementary material for: Cancer risk in individuals with intellectual disability in Sweden: A population-based cohort study
Source: PLoS Med. 2021 Oct 21;18(10):e1003840. doi: 10.1371/journal.pmed.1003840 (PMC8568154; doi:10.1371/journal.pmed.1003840)
Supplement: S1 Text. Analysis plan — (PDF) [file pmed.1003840.s017.pdf]

Cancer risk in individuals with intellectual disability: A population-based cohort study in Sweden

Analysis plan

Qianwei Liu

Institute of Environmental Medicine, Karolinska Institutet

Stockholm, Sweden

ID and cancer—Analysis plan

Created by Qianwei Liu: 2019.06.10

Updated by Qianwei Liu: 2020.08.11

Updated by Qianwei Liu: 2021.08.21 (adding (non-prespecified) analysis according to comments from external reviewers and changes are marked as italic)

Objective

The overall aim of the study is to investigate the association between intellectual disability (ID) and cancer.

The primary objective of the study is to investigate the association between ID and risk of subsequent cancer and to test

1. Whether such association differs by severity of ID.
2. Whether such association differs between syndromic ID and idiopathic ID.
3. Whether such association change after adjusting for confounding.

Study Population

Through the individually unique personal identity number assigned to each citizen, we will identify all children born to Nordic mothers from 1974 to 2013 with close to complete follow-up for ID and cancer, by cross-linking Medical Birth Register, National Patient Register, Migration Register and Swedish Cancer Register. Individuals without information on fathers, without information on sex or with conflicting information (death, emigration, cancer diagnosis before birth) will be excluded from the analysis.

We will compile an exposed cohort of all individuals who received their first diagnosis of an intellectual disability during follow-up. As there are various factors clustering in families perhaps involving in the association between intellectual disability and cancer, we will further conduct a sibling-based comparison, in which we will compare patients with ID to their ID-free full siblings through linking to Swedish Multi-generation Register. Population reference will be defined as all others free of ID and without full siblings with ID.

Follow-up

Each live born child will be followed from birth until emigration, death, the end of follow-up (December 31, 2016) or a diagnosis of cancer, whichever came first. The follow-up of unexposed individuals or siblings was additionally censored if they were later diagnosed as having ID. These individuals will then be moved to the exposed group. The follow-up of unexposed individuals was also additionally censored if their siblings were later diagnosed as having ID. These individuals will then be moved to the sibling comparison group.

Intellectual disability

We will use Swedish revision of the International Classification of Diseases (ICD) codes to classify ID, ID severity (mild ID, moderate, severe, profound, other or unspecific ID), and ID with syndrome or without syndrome through National Patient Register.

Outcome

We will first identify cancers through linking cohort to Swedish Cancer Register, using the Swedish 7th revision of the International Classification of Diseases (ICD-7) codes. We will further study individual cancer subtypes- cancer types with at least one case among individuals with ID. A child with more than one cancer type will be counted as cancer case in the analysis of several cancer types.

Covariates

We will collect information on sex, birth year, maternal age at delivery, maternal smoking during pregnancy (at 1st antenatal visit), gestational age, birth weight, Apgar score at 1 minute, and multiple births from Medical Birth Register. We will obtain information about father-id and paternal age at delivery by crosslinking to the Swedish Multi-Generation Register. Maternal and paternal educational levels will be collected from the Swedish Longitudinal Integration Database for Health Insurance and Labor Market, as a measure of socioeconomic status. Information about parental psychiatric history and parental cancer history at offspring birth will be collected from the National Patient Register and the Swedish Cancer Register.

Statistical analysis

We will calculate crude incidence rates of cancer (cases per 100,000 person-years) and estimate hazard ratios (HRs) and associated two-sided 95% Wald-type confidence intervals. We will use attained age as the underlying time scale in all analyses and the analyses will be performed in two models for overall cancer and each cancer type. In model 1, analyses will be only adjusted for sex and birth year. To rule out that a categorical representation of birth year caused residual confounding, we will fit the models using natural cubic splines. In model 2, we will additionally adjust for maternal and paternal age, and 'inherited risk' by additionally including indicators for presence of maternal and paternal psychiatric diagnosis and maternal and paternal cancer diagnosis at delivery. We will repeat the analyses above by severity of ID - individuals with mild ID, and moderate, severe, profound, other or unspecific ID versus population controls. We will repeat the analyses stratified by idiopathic ID and syndromic ID versus population controls. Finally, we will repeat the main analyses in subgroups of males and females.

Supplementary and sensitivity analyses

We will perform a sequence of supplementary and sensitivity analyses. **(1)** To rule out that our results are due to familial confounding, we will conduct a sibling-design analysis to estimate the within-family specific cancer risk by fitting conditional Cox regression models with full-sibling families (mother-id and father-id) as strata. **(2)** We will investigate the effect of intelligence quotient (IQ) score on the association through deriving IQ score from the ICD diagnostic codes. **(3)** To further explore effect of birth characteristics, parental education and maternal smoking during pregnancy, we will adjust for gestational age (<37 weeks, 37-41 weeks, >41 weeks, (37-41 weeks as reference)), birth weight (<2.5 kg, 2.5-4 kg, > 4 kg, (2.5-4 kg as reference)), Apgar score

at 1 minute (>=7, 4-6, <=3), multiple births (yes or no), maternal and paternal education level (<9 years, 9-12 years, >12 years) and maternal smoking during pregnancy (yes or no), separately. We will also perform stratified analyses by gestational age (<37 weeks, 37-41 weeks, and >41 weeks), birth weight (<2.5 kg, 2.5-4 kg, and > 4 kg), Apgar score at 1 minute (>=7, 4-6, and <=3), multiple births or singleton, maternal education and paternal education level (<9 years, 9-12 years, >12 years) and maternal smoking during pregnancy (yes or no). **(4)** As pre-term birth is strongly related to both ID and cancer, we will repeat the main analysis in individuals with pre-term birth. **(5)** As ID might have different effect on the risk of cancer in childhood and early adulthood, we will further repeat analyses by restricting outcome as childhood cancer. **(6)** As diagnostic criteria and screening strategy for ID and cancer might have changed during the study, we will compare cancer risk among individuals born during different calendar years. **(7)** To address influence of inherited cancer risk, we will plot cancer risk by the estimated heritability of cancer. **(8)** We will plot survival curves of risk of cancer from Cox regression models adjusting for birth year and sex (model 1) to explore age-specific risks.

Non-prespecified Analysis (Analysis according to comments from external reviewers):
(1) *As the analyses of individual cancer types restricted to cancers with at least one case might introduce bias, in a sensitivity analysis, we will study cancer types by organ systems that included cancer types with or without case among individuals with ID.*

(2) *To address the possibility of reverse causation where ID may be caused by a cancer tumor, e.g., preclinical CNS cancer, we will perform an additional analysis in which individuals with CNS cancer occurring within the first five years after diagnosis of ID, or start of follow-up, were excluded.*

Software

The SAS 9.4 (SAS Institute) will be the primary analysis tool.

Statistical Considerations

Considering relatively low statistical power of some individual cancers, when analyzing some cancer subtypes, detailed adjustment for confounding will depends on the actual situation. As some individuals may have missing value on Apgar score at 1 minute, maternal smoking, maternal education and paternal education, multiple imputation will be performed for missing values.
