# Supplementary material for: Cancer risk in individuals with intellectual disability in Sweden: A population-based cohort study
Source: PLoS Med. 2021 Oct 21;18(10):e1003840. doi: 10.1371/journal.pmed.1003840 (PMC8568154; doi:10.1371/journal.pmed.1003840)
Supplement: S2 Text. SAS codes — (PDF) [file pmed.1003840.s018.pdf]

Cancer risk in individuals with intellectual disability: A population-based cohort study in Sweden

SAS codes

Qianwei Liu

Institute of Environmental Medicine, Karolinska Institutet

Stockholm, Sweden

```
*------------------------------------------------------------------------------;
* Project.....: REC1905                                                        ;
* Name........: dm_ana_pop                                                     ;
* Date........: 2020-06-12                                                     ;
* Update......: 2021-08-26                                                     ;
* Author......: Qianwei Liu                                                    ;
* Purpose.....: Create a dataset for analysis and perform main analysis        ;
* ............: Final dataset and main analysis                                ;
*------------------------------------------------------------------------------;
* OP..........: Linux/ SAS ver 9.04.01M6P110718                                ;
*------------------------------------------------------------------------------;
* Data used: P:\0RECAP\0RECAP_Research\sasproj\REC\REC1905\sasdsn              ;
* Data created:  Allcohort11 cancer_outcome1                                   ;
*------------------------------------------------------------------------------;

*-- Main program --------------------------------------------------------------;

*------------------------------------------------------------------------------;
* Build cohort for ID cases, ID-free full siblings and reference population   ;
*------------------------------------------------------------------------------;

*/removing observations with the same father id and mother id to ensure the
quality/;
data child_pop;
  set study.Rec1905_population(keep=mother_id mage fage fland father_id
                                    child_id b_yr sex plural b_dt ga_w
                                    bwt apgar1 msmok);
  if father_id=mother_id then delete;* 7 observation removed;
run;

*Limiting birth year (1974-2013);
data child_pop_00;
  set child_pop;
  if 1974<= b_yr <=2013;
run;
*A toal of 3557910 children;

*/Remove observations without father information(individuals without father_id
are the same as individuals without fland)/*;
data child_pop_01;
  set child_pop_00(where=(fland^='M'));
run;
* 23229 observations without father information, leaving 3534681 observations;


*/Remove observations without sex information from population/*;
data child_pop_02;
  set child_pop_01(where=(sex>0));
run;
*7 observations without sex information, leaving 3534674 observations;

*Sorted by child_id;
proc sort data= child_pop_02;
  by child_id;
run;
*3534674;
```

```
/* Select cancer diagnoses for the study population */;
data child_cancer;
  merge child_pop_02(in=child_pop_02 keep=child_id)
        study.Recap_cancer(in=Recap_cancer rename=(pid=child_id)
                           keep=pid DIAG_DAT_CHAR BEN ICD7);
  by child_id;

  if child_pop_02 and Recap_cancer;
run;
*69466 observations;

*Sorted by child_id and diagnostic date;
proc sort data=child_cancer;
  by child_id DIAG_DAT_CHAR;
run;
*69466 observations;

*/Dealing with observations without exact diagnosis date (by imputing day 15)
and keep only malignant cancer/*;

data child_cancer_02;
drop  DIAG_DAT_CHAR ben;
  attrib diadatn length=4 format=yymmdd10. label='cancer date';
  set child_cancer;
  if substr(ben,1,1) ^='3' then do;
  if length(compress(DIAG_DAT_CHAR))=6 then
  DIAG_DAT_CHAR=compress(DIAG_DAT_CHAR)||'15';
  else if substr(compress(DIAG_DAT_CHAR), 7, 2)='00' then
  DIAG_DAT_CHAR=substr(compress(DIAG_DAT_CHAR), 1, 6)||'15';
  diadatn = input(DIAG_DAT_CHAR, yymmdd8.);
  end;
  else delete;
run;
*27423;

*Sorted by child_id and diagnostic date;
proc sort data=child_cancer_02;
  by child_id diadatn;
run;
*27423;

*first child cancer;
proc sort data=child_cancer_02
  nodupkey out=first_cancer;
  by child_id;
run;
*26022;


*/Combination of censored events in children cohort/*;
data child_pop_03;
  merge child_pop_02(in=a)
        study.Rec1905_child_demo(in=b keep=child_id emigr_dt death_dtorig)
        first_cancer(in=c keep=child_id diadatn);
  by child_id;
  if a;
```

```
run;
*3534674;

*generate numeric death date;
data child_pop_04;
  attrib death_date length=4 format=yymmdd10. label='Date of death';
  set child_pop_03;
  death_date= input(death_dtorig, yymmdd12.);
run;
* 3534674 children;


*/ define entry and exit of cohort and exclude observations with conflicting
information/;
data birth_cohort_01;
  attrib entry length=4 format=yymmdd10. label='Date of cohort entry' ;
  attrib exit length=4 format=yymmdd10. label='Date of cohort exit' ;
  set child_pop_04;
  entry=b_dt; exit=min('31DEC2016'd, emigr_dt, death_date, diadatn);
  if exit>entry;
run;
*3,369 individuals with conflicting information (death, emigration, cancer
diagnosis before birth)
, leaving 3531305 children;


*---------------------------;
* Definition of 1.ID cohort;
*---------------------------;

*/Defining exposure group/;
* exposure: 0 represents Individuals without ID and without siblings of ID, 1
represents ID patients or their siblings;
* expo_gp: 0 represents individuals without ID and without siblings of ID, 1
represents ID patients, 2 represents siblings of ID;

*Exposure cohort;
data exposure_cohort_01;
  length exposure expo_gp 3;
  attrib period_start length=4 format=yymmdd10. label='Date of actual entry of
each cohort' ;
  attrib period_end length=4 format=yymmdd10. label='Date of actual exit of
each cohort' ;
  merge birth_cohort_01(in=a)
        intellectual_disability_6(in=b keep=child_id INDATUM);
  by child_id;
  if a and b;
      exposure=1;*define exposure (exposure=0,reference);
      expo_gp=1; *define exposure group(reference:expo_gp=0, ID
      cases:expo_gp=1, ID free full-siblings:expo_gp=2);
      period_start=max(entry,INDATUM);period_end=exit;
  format period_start period_end yymmdd10.;
run;
*28382 observations;

*refining period_start<period_end;
data exposure_cohort_02;
```

```
  set exposure_cohort_01;
  if period_start<period_end;
run;
*27956 individuals with ID;

*Attach indatum of ID to birth cohort;
data  birth_cohort_02;
  merge birth_cohort_01(in=a)
        intellectual_disability_6(in=b keep=child_id indatum);
  by child_id;
  if a;
run;

*/Reference cohort1(including full-siblings of exposure group and population
control)/;
data  ref_cohort1;
  length exposure expo_gp 3;
  attrib period_start length=4 format=yymmdd10. label='Date of actual entry of
each cohort' ;
  attrib period_end length=4 format=yymmdd10. label='Date of actual exit of
each cohort' ;
  merge birth_cohort_02(in=a)
       intellectual_disability_6(in=b keep=child_id indatum);
  by child_id;
  if a;
     exposure=0; *define exposure (exposure=0,reference);
     expo_gp=0; *define exposure group(reference:expo_gp=0, ID
     cases:expo_gp=1, ID free full-siblings:expo_gp=2);
     period_start=entry;period_end=min(indatum,exit);
  format period_start period_end yymmdd10.;
run;
*3531305 observations;


*---------------------------------;
* definition of 2.sibling cohort  ;
*---------------------------------;


*Attach sibling ID to reference cohort;
data sibling1;
  merge ref_cohort1(in=a)
        index_sibling_6(in=b keep=child_id Sibling_ID);
  by child_id;
  if a;
run;
*4961002 records;

*Identify full-sibling of ID cases;
proc sql;
  create table sibling2 as
  select a.indatum,
         b.child_id,
         b.sibling_id
  from  intellectual_disability_6 as a inner join sibling1 as b
  on a.child_id=b.Sibling_ID
  order by child_id, indatum;
```

```
quit;
*34859;


*Transpose sibling's diagnostic time in one row for each child;
proc transpose data=sibling2  out=sibling3;
  by child_id;
  var indatum;
run;
*33136;

*Identify the earliest time of ID diagnosis in family for each child;
data sibling4;
  attrib family_indatum length=4 format=yymmdd10.
  label='Date of first ID in family';
  set sibling3;
  family_indatum=min(col1,col2,col3,col4,col5);
  keep child_id family_indatum;
run;
*33136;

*Check siblings of cases;
data sibling5;
  set sibling4;
  if family_indatum>.;
run;
 *33136;


*sibling cohort;
data sibling_cohort;
  length exposure expo_gp 3;
  attrib period_start length=4 format=yymmdd10.
  label='Date of actual entry of each cohort' ;
  attrib period_end length=4 format=yymmdd10.
  label='Date of actual exit of each cohort' ;
  merge ref_cohort1(in=a)
        sibling5(in=b);
  by child_id;
  if a and b;
      exposure=1; *define exposure(reference:exposure=0);
      expo_gp=2; *define exposure group(reference:expo_gp=0, ID
      cases:expo_gp=1, ID free full-siblings:expo_gp=2);
      period_start=max(period_start,family_indatum);
  format period_start period_end yymmdd10.;
run;
*33136 observations;

data sibling_cohort_01;
  set sibling_cohort;
  if period_start<period_end;
run;
*30845 ID free full siblings of ID;

*-----------------------------;
* definition of 3.reference  ;
*-----------------------------;
```

```
*/reference cohort(excluding full-siblings of ID cases)/;
data ref_cohort2;
  attrib period_end length=4 format=yymmdd10.
  label='Date of actual exit of each cohort' ;
  merge ref_cohort1(in=a)
        sibling5(in=b);
  by child_id;
  if a;
  period_end=min(family_indatum,period_end);
run;
*3531305;

data ref_cohort3;
  set ref_cohort2;
  if period_start<period_end;
run;
*3528923;


*/Allcohort including ID cohort, ID-free full siblings of ID individuals and
reference/;
data allcohort;
  set exposure_cohort_02
      ref_cohort3
      sibling_cohort_01;
run;
*3587724 observations of 3531305 children (Individuals with ID and ID-free
full siblings contribute reference period to
reference cohort before diagnosis of ID or ID diagnosis of full-siblings);

*---------------------------;
* Attach variables          ;
*---------------------------;
*First cancer of mother and father of each child;

*cancer_father;
proc sql;
  create table cancer_father as
  select a.FATHER_ID,
         b.PID,
         b.DIAG_DAT_CHAR,
         b.BEN,
         b.ICD7
         from  allcohort as a inner join study.Recap_cancer as b
  on a.FATHER_ID=b.PID
  order by FATHER_ID, DIAG_DAT_CHAR;
quit;
*361169;

*Keep only malignant cancer and dealing with missing date (by imputing day 15)
of cancer diagnosis on fathers of studied children;
data facancer1;
  drop  DIAG_DAT_CHAR ben;
  attrib fcan_date length=4 format=yymmdd10. label='father cancer date'
  ;
  set cancer_father  (keep=father_id DIAG_DAT_CHAR ben icd7 );
  if substr(ben,1,1) ^='3' then do;
```

```
  if length(compress(DIAG_DAT_CHAR))=6 then
  DIAG_DAT_CHAR=compress(DIAG_DAT_CHAR)||'15';
  else if substr(compress(DIAG_DAT_CHAR), 7, 2)='00' then
  DIAG_DAT_CHAR=substr(compress(DIAG_DAT_CHAR), 1, 6)||'15';
  fcan_date = input(DIAG_DAT_CHAR, yymmdd8.);
  end;
  else delete;
run;
*309912 observeations;

*-- Sort by ID and diagnosis date to keep the first event in the following
step;
proc sort data=facancer1  out=facancer2 ;
  by father_id fcan_date;
run;

*first diagnosis;
proc sort data= facancer2 nodupkey out=first_facancer;
  by father_id;
run;
*151446;



*cancer_mother;
proc sql;
  create table cancer_mother as
  select a.MOTHER_ID,
       b.PID,
       b.DIAG_DAT_CHAR,
       b.BEN,
       b.ICD7
  from  allcohort as a inner join study.Recap_cancer as b
  on a.MOTHER_ID=b.PID
  order by MOTHER_ID, DIAG_DAT_CHAR;
quit;
*592674;

*Keep only malignant cancer and dealing with missing date (by imputing day 15)
of cancer diagnosis on mothers of studied children;
data mocancer1;
  drop ben;
  attrib mcan_date length=4 format=yymmdd10. label='mother cancer date';
  set cancer_mother  (keep=mother_id DIAG_DAT_CHAR ben icd7 );
  if substr(ben,1,1) ^='3' then do;
  if length(compress(DIAG_DAT_CHAR))=6 then
  DIAG_DAT_CHAR=compress(DIAG_DAT_CHAR)||'15';
  else if substr(compress(DIAG_DAT_CHAR), 7, 2)='00' then
  DIAG_DAT_CHAR=substr(compress(DIAG_DAT_CHAR), 1, 6)||'15';
  mcan_date = input(DIAG_DAT_CHAR, yymmdd10.);
  end;
  else delete;
run;
*303909 observeations;

*-- Sort by ID and cancer date to keep the first event in the following step;
proc sort data=mocancer1  out=mocancer2 ;
```

```
  by mother_id mcan_date;
run;

*first cancer diagnosis;
proc sort data= mocancer2 nodupkey out=first_mocancer;
  by mother_id;
run;
*143609;

*Attach father cancer date to child;
data allcohort01 ;
  set allcohort;
run;

proc sort data=allcohort01;
  by FATHER_ID;
run;

data allcohort02;
  merge allcohort01(in=a)
        first_Facancer(in=b keep=father_ID Fcan_date);
  by father_id;
  if a;
run;
*3587724;


*Attach mother cancer date to child;
proc sort data=allcohort02;
  by MOTHER_ID;
run;

data allcohort03;
  merge allcohort02(in=a)
        first_mocancer(in=b keep=mother_ID Mcan_date);
  by mother_id;
  if a;
run;

proc sort data=allcohort03;
  by child_ID;
run;
*generate unique id for each observation;
data allcohort04;
  set allcohort03;
  length wu_id 5;
  wu_id=_n_;
run;

*Attach Mother education;
*edu: 1='less than 9 years' 2='9 to 12 years' 3='more than 12 years';

data mo_edu;
  attrib edu length=3 label='Education category';
  set study. Rec1905_motheredu(keep=Mother_ID edu_year Sun2000niva_old);
  if substr(Sun2000niva_old,1,1)='1' then edu=1;
  if substr(Sun2000niva_old,1,1) in('2','3','4') then edu=2;
```

```
  if substr(Sun2000niva_old,1,1) in('5','6','7') then edu=3;
  keep Mother_ID edu_year edu;
run;


proc sql;
  create table allcohort05  as
  select a.*,
         b.edu as mo_edu length=3 label='maternal Education'
  from allcohort04 as a left join mo_edu as b
  on a.mother_ID=b.mother_ID and a.b_yr=b.edu_year;
quit;
*3587726;


proc sort data=allcohort05
  nodupkey out=allcohort06;
  by wu_id;
run;
*3587724;


*Attach Father education (edu: 1='less than 9 years' 2='9 to 12 years' 3='more
than 12 years');
data fa_edu;
  set study. Rec1905_fatheredu(keep=father_ID edu_year Sun2000niva_old);
  if substr(Sun2000niva_old,1,1)='1' then edu=1;
  if substr(Sun2000niva_old,1,1) in('2','3','4') then edu=2;
  if substr(Sun2000niva_old,1,1) in('5','6','7') then edu=3;
  keep father_ID edu_year edu;
run;
*45569846;

proc sql;
  create table allcohort07  as
  select a.*,
         b.edu as fa_edu length=3 label='Paternal Education'
  from allcohort06 as a left join fa_edu as b
  on a.father_ID=b.father_ID and a.b_yr=b.edu_year;
quit;
*3587733;

proc sort data=allcohort07
  nodupkey out=allcohort08;
  by wu_id;
run;
*3587724;


*Attach parental psychiatric disorders to each child;
proc sql;
  create table mother_psy as
  select a.MOTHER_ID,
         b.PID,
         b.DIAG_DAT,
         b.any_psych
  from  study.rec1905_population as a inner join phychiatric_his as b
```

```
  on a.MOTHER_ID=b.PID and a.b_dt> b.diag_dat
  order by MOTHER_ID, DIAG_DAT;
quit;

*sorted by mother ID and date;
proc sort data=mother_psy out=mother_psy1 ;
  by MOTHER_ID DIAG_DAT;
run;

*remove duplicates;
proc sort data= mother_psy1 nodupkey out=mother_psy2;
  by MOTHER_ID;
run;

data mother_psy3;
  set mother_psy2;
  rename any_psych=m_psy;
run;

proc sql;
  create table father_psy as
  select a.faTHER_ID,
         b.PID,
         b.DIAG_DAT,
         b.any_psych
  from study.rec1905_population as a inner join phychiatric_his as b
  on a.faTHER_ID=b.PID and a.b_dt> b.diag_dat
  order by faTHER_ID, DIAG_DAT;
quit;

*sorted by father ID and date;
proc sort data=father_psy  out=father_psy1 ;
  by faTHER_ID DIAG_DAT;
run;

*remove duplicates;
proc sort data= father_psy1 nodupkey out=father_psy2;
  by faTHER_ID;
run;

data father_psy3;
  set father_psy2;
  rename any_psych=f_psy;
run;

*Attache maternal psychiatric information;
proc sql;
  create table allcohort09 as
  select a.*,
         b.m_psy
  from allcohort08 as a left join  mother_psy3 as b
  on a.mother_ID=b.mother_ID;
quit;

*Attache paternal psy;
proc sql;
  create table allcohort10  as
```

```
  select a.*,
         b.f_psy
  from allcohort09 as a left join  father_psy3 as b
  on a.father_id=b.father_ID;
quit;

proc sort data=allcohort10;
  by child_id;
run;



*/Defining Categories of continuous variables/;
data  allcohort11;
  set allcohort10(rename=(m_psy=mpsy) rename=(f_psy=fpsy));
  age=ceil((period_start-b_dt)/365.24);
  if age<=5 then agegp=1;
  if 5<age<=10 then agegp=2;
  if 10<age<=15 then agegp=3;
  if 15<age<=20 then agegp=4;
  if 20<age then agegp=5;
  if 1974<=b_yr<=1983 then byr_gp=1;
  if 1984<=b_yr<=1993 then byr_gp=2;
  if 1994<=b_yr<=2003 then byr_gp=3;
  if 2004<=b_yr<=2013 then byr_gp=4;
  if mage< 20 then mage_gp=1;
  if 20<=mage<=29 then mage_gp=2;
  if 30<=mage<=39 then mage_gp=3;
  if 40<=mage then mage_gp=4;
  if mage=. then mage_gp=.;
  if bwt< 2500 then bwt_gp=1;
  if 2500<=bwt<=4000 then bwt_gp=2;
  if bwt>4000 then bwt_gp=3;
  if '01'<=substr(APGAR1,1,2)<='03' then apgar_gp=1;
  if '04'<=substr(APGAR1,1,2)<='06' then apgar_gp=2;
  if '10'>=substr(APGAR1,1,2)>='07' then apgar_gp=3;
  if ga_w<37 then ga_gp=1;
  if 37<=ga_w<=41 then ga_gp=2;
  if ga_w>41 then ga_gp=3;
  if fage< 20 then fage_gp=1;
  if 20<=fage<=29 then fage_gp=2;
  if 30<=fage<=39 then fage_gp=3;
  if 40<=fage then fage_gp=4;
  if fage=. then fage_gp=.;
  if .<Mcan_date<=b_dt then Mcan=1;else Mcan=0;
  if .<Fcan_date<=b_dt then Fcan=1;else Fcan=0;
  if mpsy=. then mpsy=0;
  if fpsy=. then fpsy=0;
run;

*3587724;

*delete datasets to save space;
proc datasets lib=work mt=data nolist;
  delete allcohort allcohort01 allcohort02 allcohort03 allcohort04 allcohort05
         allcohort06 allcohort07 allcohort08 allcohort09 allcohort10
         Birth_cohort_01 Birth_cohort_02 Cancer_father Cancer_mother
```

```
         Child_cancer Child_cancer_01 Child_pop Child_pop_01 Child_pop_02
         Child_pop_03 Child_pop_04 Exposure_cohort_01 Exposure_cohort_02
         Facancer1 Facancer2 Fa_edu Mo_edu First_facancer
         First_mocancer Mocancer1 Mocancer2 Ref_cohort1 Ref_cohort2
         Ref_cohort3 sibling1 sibling2 sibling3 sibling4 sibling5
         Sibling_cohort Sibling_cohort_01;

quit;



*Attach cancer outcome to cohort;

*Redifinition of period end to allow multiple cancer outcomes ;
data entire_cohort;
  attrib period_end1 length=4 format=yymmdd10.
  label='End of follow-up(not considering cancer diagnosis)';
  set Allcohort11;
  if expo_gp=0 then period_end1=min('31DEC2016'd, emigr_dt, death_date,
     indatum, FAMILY_INDATUM);
  else if expo_gp=1 then period_end1=min('31DEC2016'd, emigr_dt, death_date);
  else if expo_gp=2 then period_end1=min('31DEC2016'd, emigr_dt, death_date,
          indatum);
  keep child_id b_yr B_DT ENTRY exit EXPOSURE--period_start MPSY SEX FPSY
      MAGE_GP FAGE_GP FCAN MCAN period_end1;
  length EXPOSURE EXPO_GP MPSY FPSY MAGE_GP FAGE_GP FCAN MCAN 3.;
  if expo_gp^=2;
run;

proc sort data=entire_cohort;
  by child_id;
run;
*3556879;

*outcome of individual cancer subtype;
data salivary_cancer oesophagus_cancer Stomach_cancer smallintestine_cancer
     Colon_cancer rectum_cancer liver_cancer pancreas_cancer lung_cancer
     Breast_cancer cervix_cancer corpus_cancer Ovary_cancer Testis_cancer
     thyroid_cancer ALL AML HL NHL kidney_cancer nonmelanoma_cancer
     melanoma_cancer eye_cancer CNS_cancer endocrine_cancer bone_cancer
     connective_cancer other_cancer;
  length outcome $20.;
  keep CHILD_ID icd7 diadatn group outcome;
  set child_cancer_02;
  if substr(ICD7,1,3)='142' then do;
  group=2; outcome='salivary';
  output Salivary_cancer;
end;

  else if substr(ICD7,1,3)='150' then do;
  group=3; outcome='oesophagus';
  output oesophagus_cancer;
end;

  else if substr(ICD7,1,3)='151' then do;
  group=4; outcome='stomach';
  output Stomach_cancer;
```

```
end;

  else if substr(ICD7,1,3)='152' then do;
  group=5; outcome='smallintestine';
  output smallintestine_cancer;
end;

  else if substr(ICD7,1,3)='153' then do;
  group=6; outcome='colon';
  output Colon_cancer;
end;

  else if substr(ICD7,1,3)='154' then do;
  group=7; outcome='rectum';
  output rectum_cancer;
end;

else if substr(ICD7,1,3)='155' then do;
  group=8; outcome='liver';
  output liver_cancer;
end;

else if substr(ICD7,1,3)='157' then do;
  group=9; outcome='pancreas';
  output pancreas_cancer;
end;

else if substr(ICD7,1,3)='162' then do;
  group=10; outcome='Lung';
  output lung_cancer;
end;

else if substr(ICD7,1,3)='170' then do;
  group=11; outcome='Breast';
  output breast_cancer;
end;

else if substr(ICD7,1,3)='171' then do;
  group=12; outcome='cervix';
  output cervix_cancer;
end;

else if '172'<=substr(ICD7,1,3)='174' then do;
  group=13; outcome='corpus';
  output corpus_cancer;
end;


else if substr(ICD7,1,3)='175' then do;
  group=14; outcome='Ovary';
  output ovary_cancer;
end;

else if substr(ICD7,1,3)='178' then do;
  group=15; outcome='Testis';
  output testis_cancer;
end;
```

```
else if substr(ICD7,1,3)='180' then do;
  group=16; outcome='kidney';
  output kidney_cancer;
end;

else if substr(ICD7,1,3)='190' then do;
  group=17; outcome='melanoma';
  output melanoma_cancer;
end;

else if substr(ICD7,1,3)='191' then do;
  group=18; outcome='Non-melanoma skin';
  output nonmelanoma_cancer;
end;

else if substr(ICD7,1,3)='192' then do;
  group=19; outcome='eye';
  output eye_cancer;
end;

else if substr(ICD7,1,3)='193' then do;
  group=20; outcome='CNS';
  output CNS_cancer;
end;

else if substr(ICD7,1,3)='194' then do;
  group=21; outcome='thyroid';
  output thyroid_cancer;
end;

else if substr(ICD7,1,3)='195' then do;
  group=22; outcome='endocrine';
  output endocrine_cancer;
end;

else if substr(ICD7,1,3)='196' then do;
  group=23; outcome='bone';
  output bone_cancer;
end;

else if substr(ICD7,1,3)='197' then do;
  group=24; outcome='connective';
  output connective_cancer;
end;

else if substr(ICD7,1,3)='199' then do;
  group=25; outcome='Other';
  output other_cancer;
end;

else if substr(ICD7,1,3)='201' then do;
  group=26; outcome='HL';
  output HL;
end;
```

```
else if substr(ICD7,1,3) in ('200','202') or substr(ICD7,1,4) in ('2041') then
do;
  group=27; outcome='NHL';
  output NHL;
end;

else if substr(ICD7,1,4) in ('2040','2049') then do;
  group=28;outcome='ALL';
  output ALL;
end;

else if substr(ICD7,1,4) in ('2050','2059','2060','2069') then do;
  group=29; outcome='AML';output AML;
end;
run;

*Any cancer;
data all_cancer;
  length outcome $20.;
  set child_cancer_02;
  keep CHILD_ID icd7 diadatn group outcome;
  group=1;
   outcome='overall';
run;

*First observation of each cancer group;
%macro cohort(out,name);
proc sort data=&out;
  by child_id diadatn;
run;

proc sort data=&out nodupkey ;
  by child_id;
run;

%mend;

%cohort(out=all_cancer);
%cohort(out=salivary_cancer);
%cohort(out=oesophagus_cancer);
%cohort(out=Stomach_cancer);
%cohort(out=smallintestine_cancer);
%cohort(out=Colon_cancer);
%cohort(out=rectum_cancer);
%cohort(out=liver_cancer);
%cohort(out=pancreas_cancer);
%cohort(out=lung_cancer);
%cohort(out=Breast_cancer);
%cohort(out=cervix_cancer);
%cohort(out=corpus_cancer);
%cohort(out=Ovary_cancer);
%cohort(out=Testis_cancer);
%cohort(out=kidney_cancer);
%cohort(out=melanoma_cancer);
%cohort(out=nonmelanoma_cancer);
%cohort(out=eye_cancer);
%cohort(out=CNS_cancer);
```

```
%cohort(out=thyroid_cancer);
%cohort(out=endocrine_cancer);
%cohort(out=bone_cancer);
%cohort(out=connective_cancer);
%cohort(out=other_cancer);
%cohort(out=HL);
%cohort(out=NHL);
%cohort(out=ALL);
%cohort(out=AML);



*Attach cancer outcome to studied cohort;
%macro cohort(out,name);
data &out;
  attrib name length=3 label='group';
  merge entire_cohort(in=a)
        &out(in=b keep=child_id diadatn);name=&name;
  by child_id;
  if a;
  drop expo_gp;
run;

%mend;

%cohort(out=all_cancer, name=1);
%cohort(out=salivary_cancer, name=2);
%cohort(out=oesophagus_cancer, name=3);
%cohort(out=Stomach_cancer, name=4);
%cohort(out=smallintestine_cancer, name=5);
%cohort(out=Colon_cancer, name=6);
%cohort(out=rectum_cancer, name=7);
%cohort(out=liver_cancer, name=8);
%cohort(out=pancreas_cancer, name=9);
%cohort(out=lung_cancer, name=10);
%cohort(out=Breast_cancer, name=11);
%cohort(out=cervix_cancer, name=12);
%cohort(out=corpus_cancer, name=13);
%cohort(out=Ovary_cancer, name=14);
%cohort(out=Testis_cancer, name=15);
%cohort(out=kidney_cancer, name=16);
%cohort(out=melanoma_cancer, name=17);
%cohort(out=nonmelanoma_cancer, name=18);
%cohort(out=eye_cancer, name=19);
%cohort(out=CNS_cancer, name=20);
%cohort(out=thyroid_cancer, name=21);
%cohort(out=endocrine_cancer, name=22);
%cohort(out=bone_cancer, name=23);
%cohort(out=connective_cancer, name=24);
%cohort(out=other_cancer, name=25);
%cohort(out=HL, name=26);
%cohort(out=NHL, name=27);
%cohort(out=ALL, name=28);
%cohort(out=AML, name=29);


*Definition of period end and outcome event;
```

```
data cancer_outcome(compress=yes) ;
  attrib period_end2 length=4 format=yymmdd10.
  label='End of follow-up(including cancer diagnosis)';
  attrib k length=3 label='Sex';
  set all_cancer
      salivary_cancer
      oesophagus_cancer
      Stomach_cancer
      smallintestine_cancer
      Colon_cancer
      rectum_cancer
      liver_cancer
      pancreas_cancer
      lung_cancer
      Breast_cancer
      cervix_cancer
      corpus_cancer
      Ovary_cancer
      Testis_cancer
      kidney_cancer
      melanoma_cancer
      nonmelanoma_cancer
      eye_cancer
      CNS_cancer
      thyroid_cancer
      endocrine_cancer
      bone_cancer
      connective_cancer
      other_cancer
      HL
      NHL
      ALL
      AML;

  k=sex;

  *-- if diagnosis is within follow-up interval (cohort entry to cohort exit);
  if period_start<=diadatn<=period_end1 then failure=1;
  else failure=0;
  period_end2=min(period_end1,diadatn);
  drop sex diadatn period_end1;

run;


*Delete datasets to save space;
proc datasets lib=work mt=data nolist;
  delete all_cancer
         salivary_cancer
         oesophagus_cancer
         Stomach_cancer
         smallintestine_cancer
         Colon_cancer
         rectum_cancer
         liver_cancer
         pancreas_cancer
         lung_cancer
```

```
         Breast_cancer
         cervix_cancer
         corpus_cancer
         Ovary_cancer
         Testis_cancer
         kidney_cancer
         melanoma_cancer
         nonmelanoma_cancer
         eye_cancer
         CNS_cancer
         thyroid_cancer
         endocrine_cancer
         bone_cancer
         connective_cancer
         other_cancer
         HL
         NHL
         ALL
         AML

  ;
quit;

*Generate age at start of follow-up and age at end of follow-up;
data cancer_outcome1;
  set cancer_outcome;
  length  b_yr period_start period_end2 4.;
  Tstart=floor((period_start-entry)/365.24);
  Tstop=floor((period_end2-entry)/365.24);
  length  EXPOSURE MPSY FAILURE Tstart Tstop 3. ;
  drop entry exit b_dt;
run;

proc datasets lib=work mt=data nolist;
  delete cancer_outcome;
quit;


*Descriptive statistics;
*Table 1 and Table S4;
Title 'table1 and s4';
*generate different groups: ID, ID siblings and reference;
*exposure reprents individuals with ID and ID free full siblings of ID.
exposure1: individuals with ID, exposure2: ID free full siblings of ID;
data reference exposure exposure1 exposure2;
  set allcohort11;
  if exposure=0 then output reference;
  if exposure=1 then output exposure;
  if expo_gp=1 then output exposure1;
  if expo_gp=2 then output exposure2;
run;

*exclude exposure period from reference;
data reference1;
  merge reference(in=a)
        exposure(in=b keep=child_id);
  by child_id;
```

```
  if a and ^b;
run;

data exposure2a;
  merge exposure2(in=a)
        exposure1(in=b keep=child_id);
  by child_id;
  if a and ^b;
run;

data allcohort12;
  set reference1
      exposure1
      exposure2a;
run;

*family size;
data sibling_id;
  set index_sibling_6(keep=child_id);
  by child_id;
  if first.child_id then count=1;
  count+1;
  if last.child_id then output;
run;

proc sort data=allcohort12;
  by child_id;
run;

*Merge studied cohort and their full_siblings;
data fasize01;
  merge Allcohort12(in=a keep=child_id expo_gp) sibling_id(in=b);
  by child_id;
  if a;
run;

*Count=. means no full-siblings;
data fasize02;
  set fasize01;
  if count=. then count=1;
  if count>2 then count=3;
run;

proc sort data=fasize02;
  by expo_gp;
run;

*Family size by exposed level;
proc freq data=fasize02;
  by expo_gp; table count / list missprint;
  format expo_gp expogpfmt.;
run;


*Summary number and proportion;

proc sort data=Allcohort12;
```

```
  by expo_gp;
 run;

 proc freq data=Allcohort12;
  by expo_gp;table sex / out=t1 list missprint;
  by expo_gp;table byr_gp  / out=t2  list missprint;
  by expo_gp;table mo_edu/ out=t3  list missprint; format mo_edu edu_fm.;
  by expo_gp;table fa_edu / out=t4 list missprint; format fa_edu edu_fm.;
  by expo_gp;table mage_gp / out=t5 list missprint;
  by expo_gp;table fage_gp/ out=t6 list missprint;
  by expo_gp;table ga_gp  / out=t7 list missprint;
  by expo_gp;table bwt_gp / out=t8 list missprint;
  by expo_gp;table apgar_gp / out=t9 list missprint;
  by expo_gp;table Msmok / out=t10 list missprint;
  by expo_gp;table PLURAL / out=t11 list missprint;
  by expo_gp;table Mpsy / out=t12 list missprint;
  by expo_gp;table Fpsy / out=t13 list missprint;
  by expo_gp;table Mcan / out=t14 list missprint;
  by expo_gp;table Fcan / out=t15 list missprint;
  format expo_gp expogpfmt.;
run;


*Generate table 2;
Title 'table2';

*Calculating Hazard ratio;
*Model 1 (adjust for birth year(natural cubic spline) and sex);
ods output hazardratios=hz1;
proc phreg data=cancer_outcome1 fast;
  where TStart < TStop;
  class exposure(ref='Individuals without ID') k(ref='1')   / order=internal;
  effect birspl = spline( b_yr / naturalcubic );
  model (TStart,TStop) * failure(0) = exposure k birspl
  /  risklimits=wald alpha=0.05;
  hazardratio exposure  /  cl=wald diff=ref;
  by name;
  format exposure exfmt.;
  format name namefmt.;
run;


*Model 2 (adjust for birth year(natural cubic spline), sex parental age,
parental history of cancer and psychiatric disorders);
ods output hazardratios=hz2;
proc phreg data=cancer_outcome1 fast ;
  where TStart < TStop;
  class exposure(ref='Individuals without ID') k(ref='1')  MPSY(ref='0')
        FPSY(ref='0') FCAN(ref='0') MCAN(ref='0') MAGE_GP(ref='2')
        FAGE_GP(ref='2') / order=internal;
  effect birspl = spline( b_yr / naturalcubic );
  model (TStart,TStop) * failure(0) = exposure k  MPSY FPSY FCAN MCAN MAGE_GP
        FAGE_GP birspl
  /  risklimits=wald alpha=0.05;
  hazardratio exposure  /  cl=wald diff=ref;
  by name;
  format exposure exfmt.;
```

```
  format name namefmt.;
run;


*Keep two decimals for hazard ratios in model 1 and model 2;
data hz1a;
  set hz1;
  HR1 = strip(put(HAZARDRATIO,6.2))||'  ('||strip(put(WALDLOWER,6.2))||'-
'||strip(put(WALDUPPER,6.2))||')';
  keep name HR1;
run;

data hz2a;
  set hz2;
  HR2 = strip(put(HAZARDRATIO,6.2))||'  ('||strip(put(WALDLOWER,6.2))||'-
'||strip(put(WALDUPPER,6.2))||')';
  keep name HR2;
run;



*Generate duration of follow-up and calculate crude Incidence rate;
data ca;
  set cancer_outcome1(keep=period_start period_end2 failure exposure name
  child_id);
  dur=(period_end2-period_start)/365.24;
  length dur 4.;
  drop period_end2 period_start;
run;

*Calculate Incidence rate by exposed level;
proc summary data=ca nway;
  var failure dur;
  class exposure;
  by name;
  output out=tmp1(drop=_type_) sum= ;
run;

data tmp2;
  set tmp1;
  x=100000*failure/dur;
run;

*Incidence rate of unexposed individuals;
data tmp3;
  set tmp2;
  y=put(x,6.2);
  if exposure=0;
run;

*Incidence rate of exposed individuals;
data tmp4;
  set tmp2;
  y1=put(x,6.2);
  if exposure=1;
run;
```

```
*Merge incidence rate and hazard ratio in one dataset;
data x;
  retain name y y1 HR1 HR2;
  merge hz1a hz2a tmp3 tmp4;
  by name;
  keep name y y1 HR1 HR2;
run;


*Print results of table 2;
proc print data=x (drop=name) noobs;run;

*-- End of File --------------------------------------------------------------
;
```
