# Supplementary material for: Cancer risk in individuals with intellectual disability in Sweden: A population-based cohort study
Source: PLoS Med. 2021 Oct 21;18(10):e1003840. doi: 10.1371/journal.pmed.1003840 (PMC8568154; doi:10.1371/journal.pmed.1003840)
Supplement: Attachment — Submitted filename: Response to Reviewers.pdf [file pmed.1003840.s019.pdf]

Requests from the editors:

Comment: Please adapt the title so that it contains a study descriptor after a colon, e.g., "Cancer risk in individuals with intellectual disability: A population-based cohort study".

Response:
Thanks for the suggestion. We have now adapted the title to "Cancer risk in individuals with intellectual disability: A population-based cohort study".

Comment: Please adapt the abstract to a three-part structure.

Response:
We have now adapted the abstract to three-part structure: Background, Methods and findings, and Conclusion (Pages 2-3).

Comment: Rather than "Nordic mothers", please write "... of mothers born in ..." or similar, in the abstract and any other instances in the paper.

Response:
We have now replaced the word "Nordic mothers" with "mothers born in a Nordic country" throughout the manuscript.

Comment: In the abstract and throughout the paper, please quote p values alongside 95% CI, where available.

Response:
We have now added p values alongside 95% CI throughout the manuscript.

Comment: Please add a final sentence to the new "Methods and findings" subsection of your abstract, which should begin "Study limitations include ..." or similar and should quote 2-3 of the study's main limitations.

Response:
We have added study limitations in the "Methods and findings" subsection of Abstract (Page 3, Lines 46-49) as follows:

"*Despite the large sample size and high-quality data, the main limitation of this study is the limited statistical power for the analysis of specific cancer types. There is a potential for underestimation of the studied association (e.g., due to potential under-detection or delayed diagnosis of cancer among individuals with ID).*"

Comment: After the abstract, we will need to ask you to add a new and accessible "Author summary" section in non-identical prose. You may find it helpful to consult one or two recent research papers in PLOS Medicine to get a sense of the preferred style.

Response:
We have now added the following "Author summary" as the editors suggested (Page 4):

"***Author summary***

Why was this study done?

Only few population-based studies have examined the association between intellectual disability (ID) and cancer risk, and these studies are, in general, hampered by limited sample size and lack of adjustment for potential confounders, including familial confounding.

What did the researchers do and find?

We conducted a population-based cohort study including more than 3.5 million Swedish children born from 1974 to 2013, to examine the association between ID and cancer.

We observed an increased risk of any cancer as well as several specific cancer types among individuals with ID.

What do these findings mean?

Considering barriers in accessing health care and the vulnerability of individuals with ID, our findings could be important for surveillance and early intervention of cancer among individuals with ID."

Comment: Early in the Methods section, please state whether or not the study had a protocol or prespecified analysis plan, and if so attach the relevant document as a supplementary file, referred to in the text.

Response:
The study had a prespecified analysis plan which has now been submitted as a supplementary file and referred to in the Methods section (Page 7, Line 124):

"*The prespecified analysis plan is presented in the* ***S1 Text.***"

Comment: Please highlight analyses that were not prespecified

Response:
We have now highlighted analyses that were not prespecified in the analysis plan, which include mainly the analyses proposed by the reviewers.

Comment: Please remove the information on funding and competing interests from the Acknowledgements section at the end of the main text. In the event of publication, this information will be included in the article metadata, via entries in the submission form.

Response:
We have now removed this information from the Acknowledgements section.

Comment: Throughout the text, please adapt reference call-outs to the following style: "... study rare cancers [11,12]." (noting the absence of spaces within the square brackets).

Response:
We have now adapted reference to the guided style.

Comment: Please abbreviate journal names consistently in your reference list.

Response:
We have now abbreviated journal names consistently in the reference list.

Comment: Where appropriate, 6 author names should be listed rather than 3, followed by "et al.".

Response:
We have now formatted author names in the reference list as guided.

Comment: Please add a completed checklist for the most appropriate reporting guideline, e.g., STROBE, as a supplementary file, labelled "S1_STROBE_Checklist" or similar and referred to as such in the Methods section.

Response:
We have now added a STROBE checklist as a supplementary file and referred to it in the Methods section (Pages 11-12, Lines 231-233):

"*This study is reported according to the Strengthening the Reporting of Observational Studies in Epidemiology (STROBE) guideline (**S3 Text**).*"

Comment: In the checklist, please refer to individual items by section (e.g., "Methods") and paragraph number, not by line or page numbers as these generally change in the event of publication.

Response:
We have referred to individual items by section and paragraph numbers in the STROBE checklist as guided.

Comments from the individual reviewers:

*** Reviewer #1:

Comment: The study titled "Cancer risk in individuals with intellectual disability" is one of its kind in both the terms of topic selection as well as the large sample size that is evaluated in the study. It strives to create a association between intellectual disability and occurrence of any form of cancer. The study would have an impact in the guidelines of management of all forms of intellectual disability in the form of adding need of cancer screening in virtually all causes of intellectual disability. It might even cause a debate or ethical dilemma regarding the management and prognosis of intellectual disability.

The strength of the study lies in the fact that:
1. The study is prospective in nature
2. The sample size is large
3. No population under study is left out: The authors claim that the children are screened and diagnosed early as well as the cancer registry is nearly encompasses all of the malignancies that might occur
4. Statistical analysis done robustly to remove certain confounding factors

Response:
Thank you for the very encouraging comments.

However certain facts cannot be overlooked while going through the study.

Comment 1. The "Introduction" section does not justify the caliber of the study or the journal. The section glances over the statistics regarding intellectual disability and malignancies. However, the causes of known and unknown causes of intellectual malignancies seem to be missing. The literature review seems to miss multiple similar papers. The scientifically proven causation of different forms of intellectual disabilities along with mutations attributed to malignancy development are not mentioned.

Response:
Thank you for the good suggestion. We have now modified the Introduction as the reviewer suggested.

Regarding causes of ID (Page 5, Lines 80-82):

"*The underlying causes of ID are heterogenous, including chromosomal abnormality, gene mutation, environmental factors and prenatal factors [7]. The exact cause is however not identifiable for most individuals with ID [8].*"

Regarding potential mechanisms linking together ID and cancer (Page 5, Lines 84-90):

"*Emerging evidence has suggested a plausible link between ID and cancer through several potential mechanisms. One possibility is that the chromosomal abnormalities or genetic mutations causing ID, especially syndromic ID, might also contribute to oncogenesis [10-12]. Another possibility is that individuals with ID may be more likely to be exposed to potential risk factors for cancer, such as unhealthy lifestyle including less optimal diet and lack of*

physical activity, which might also contribute to the initiation or development of some cancers [13,14]."

Regarding additional references as suggested by the reviewer (Pages 5-6, Lines 91-99):

"*However, population-based studies examining the association between ID and cancer are largely missing. Earlier clinical studies have suggested an increased risk of some specific types of cancer among individuals with genetic syndromes known to be associated with ID, such as Down syndrome, Fragile X syndrome, and tuberous sclerosis complex [15-17]. There is however no consensus so far about the risk of cancer in general among individuals with syndromic ID [3,18-22]. In addition, as previous studies have predominantly focused on syndromic ID, little is known about cancer risk among individuals with any ID, especially idiopathic ID.*"

"Among the few studies that studied any ID, most used cancer death or specific cancer types as the outcome of interest *[23-27]*."

Comment 2. The "Methodology" section establishes the fact that cases under study are unlikely to be missed. However, not all forms of intellectual disabilities manifest at a young age. The authors mention about a mandatory wellness child examination at the age of four. They fail to mention where the examination occurs. For instance if it occurs in school, would the children at home, which is likely in intellectually disabled child due to logistic or other reasons, be missed?

Response:
The mandatory development assessment is conducted in local child health centers. In the present study, we identified ID cases through clinical diagnoses according to the Swedish Patient Register. Because this register has nationwide coverage for hospital care for psychiatric disorders in Sweden (from 1973 for inpatient care and from 2001 for both inpatient and outpatient care), we should have identified all ID cases with a specialist-based clinical diagnosis, both at young and old ages. The reviewer is however correct that this ascertainment method could have missed some cases of ID, given the specialist-care based nature of the data and the lack of outpatient data before 2001.

We have now clarified the place where the mandatory development assessment is conducted in the manuscript (Page 7, Lines 128-136):

"*In Sweden, primary health care service for children was provided by child welfare centres to support health and development of children from infancy to 6 years of age, and nearly 100% of the children participate in the follow-up visits [34]. As part of the Swedish child health care program, routine medical and developmental screening is regularly conducted for all neonates and preschool children. A more detailed developmental assessment (motor, language, cognitive, and social development) is done at 2.5 and 4 years of age. Children with a suspected ID or other developmental abnormality are referred to a specialized team of pediatricians and child psychologists for further investigation.*"

We have also stressed the limitation in Discussion (Page 15, Lines 302-304):

"No access to ID diagnosis from primary care and inclusion of outpatient diagnoses in the National Patient Register only from 2001 might potentially have led to under-detection of ID cases, especially mild ID."

Comment 3. In the "Statistical analysis" section, does the comparison of IQ score with risk of cancer have any clinical significance?

Response:
As individuals with ID usually take IQ test, it could be important for clinicians or family members to know whether IQ score impacts the association between ID and cancer risk. In this study, the analysis of IQ score complements the analysis by ID severity. We have clarified the purpose of this analysis in Statistical analysis (Page 10, Lines 204-207):

*"As individuals with ID usually take intelligence quotient (IQ) test, it could be important to know whether IQ score impacts the association between ID and cancer risk. We derived IQ score from the ICD codes and examined the association between IQ and cancer risk (**S1 Table**)."*

Comment 4. The "Discussion" portion of the study does not compare multiple similar articles.

Response:
We have now compared all previous studies, including prevalence of ID by sex and risk of individual cancer types, in Discussion:

Pages 15-16, Lines 320-324:

"In line with previous research [46], we observed a higher prevalence of ID among males than females. One potential reason is that males have higher vulnerability of X-chromosome linked syndrome, such as fragile X syndrome, which is related to higher risk of ID [47]. Another reason might be differential brain development due to different androgen exposure between males and females [48]."

Page 16, Lines 338-343:

"Our study showed increased risks for colon and uterus cancers in females, CNS cancer in males, as well as leukemia in both males and females, among individuals with ID, which is in line with one previous study [29]. However, we did not observe statistically significantly increased risk of stomach cancer or decreased risk of prostate cancer among males [29] or increased risk of gallbladder and thyroid cancers in general [28], among individuals with ID. The contrasting results may be partly due to different age ranges of studied participants between different studies."

Further, there are also a few articles examining the association between ID and cancer death or between certain types of syndromic ID and cancer. Because of the relatively different research questions addressed in these studies, compared to our study, we decided not to compare our findings with that of these previous articles. We are however willing to reconsider our position if the reviewer and editors would disagree with us.

Comment: Ways to improve the article:

Comment 1. The authors need to perform literature review and update the "Introduction" and "Discussion" section.

Response:
We have now performed a new literature review and updated the "Introduction" and "Discussion" sections. Please see our responses to the comments 1 and 4 above.

Comment 2. If they have sufficient data from the registry, could they demonstrate the occurrences of proven malignancies to proven clinical diagnoses. e.g. prevalence of certain malignancies in Down syndrome. This would further validate the data.

Response:
Thank you for the suggestion. However, risk of cancer in Down syndrome is well established and has been examined earlier in the Swedish population using Swedish Patient Register and Swedish Cancer Register, including a study by one of our co-authors (Hans-Olov Adami) [Hill DA, Gridley G, Cnattingius S, Mellemkjaer L, Linet M, Adami HO, et al. Mortality and cancer incidence among individuals with Down syndrome. Arch Intern Med. 2003;163(6):705-11. Epub 2003/03/18. PubMed PMID: 12639204]. Since the research aim of our study was to examine cancer risk in ID but not Down Syndrome, the data we ordered from the National Register do not allow us to distinguish Down Syndrome from other syndromes that are related to ID.

Comment 3. Was the family history of malignancy collected? The manuscript does not explicitly mention this. If so, was the "end point" of malignancy detection in the intellectually disabled individuals the same as for family members.

Response:
Family history of malignancy was indeed collected. We have now clarified this in Methods (Page 9 Lines 168-170):

"*Information on parental history of psychiatric disorders or cancer at delivery was collected at birth of the cohort participants according to the National Patient Register and the Cancer Register, respectively (ICD codes listed in **S1 Table and S3 Table**).*"

Comment 4. The article needs overall editing of the language for it to be more comprehensible. Maybe technical terms could be better phrased so that it would find a wider audience.

Response:
Thank you for the suggestion. We have now revised the manuscript to avoid the use of jargon with the aim to facilitate the reading by a wider audience.

5. Could the authors mention also about the diagnosed causes of intellectual disability according to prevalence?

Response:
We have now shown this information in Results (Page 12, Lines 238-239):

"*Among individuals with ID, 9,878 had syndromic ID (35.3%) whereas 18,078 had idiopathic ID (64.7%).*"

Comment 6. The "Methodology" section could be overhauled to make it easier for the study to be reproduced.

Response:
We have extended the Methods section, including adding a flow chart to illustrate the buildup of the cohort (Supplementary S1 Fig.) and uploading a prespecified analysis plan and the SAS codes as supplementary materials.

In Methods (Page 7, Lines 121-124):
"*We first identified a total of 3,557,910 individuals in the study cohort* ***(S1 Fig)****. Individuals with no information on father (N=23,229), no information on sex (N=7), or inconsistent information (emigration, death, or cancer diagnosis before birth, N=3,369), were excluded from the analysis. The prespecified analysis plan is presented in the* ***S1 Text****.*"

In Methods (Page 11, Lines 229-230):
"*The SAS codes for data management and main analysis are presented in* ***S2 Text****.*"

Comment 7. Compare the malignancy trend with the trend in the country. Does it reflect the prevalence/incidence of malignancies as evidenced by other studies or cancer registry?

Response:
We compared our data on cancer to the ones provided by Statistics Sweden (SCB) (https://sdb.socialstyrelsen.se/if_can/val_eng.aspx), which has detailed and nationwide complete information on number and incidence of each cancer type by age, sex, calendar year and region in Sweden. Our data are largely comparable to the national statistics shown by the SCB.

Comment 8. Present the data in a more palatable graphical representations instead of only tables.

Response:
Thanks for the good suggestion. We have now used S3 Fig to replace Table 3, and used Fig 1 to replace Table 4 (see below):

S3 Fig. Intellectual disability (ID) severity specific hazard ratios (HRs) of cancer among individuals with ID by cancer type, compared to reference group.

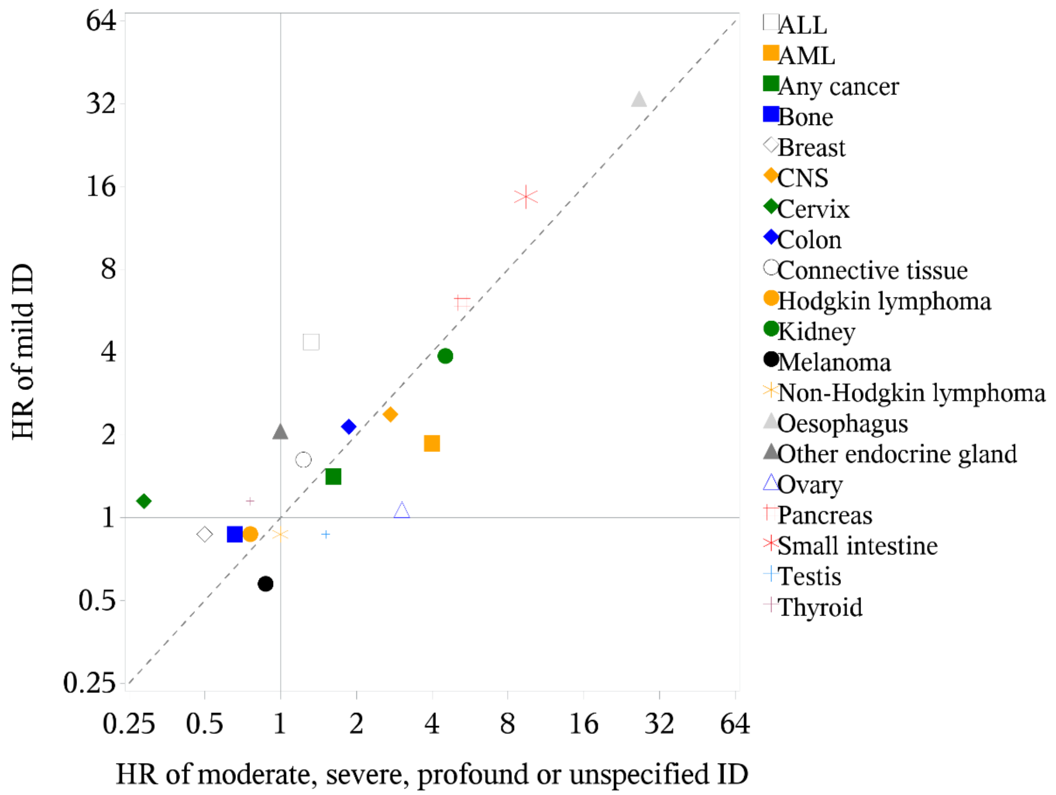

Fig 1. Intellectual disability (ID) type specific hazard ratios (HRs) of cancer among patients with ID by cancer type, compared to reference group

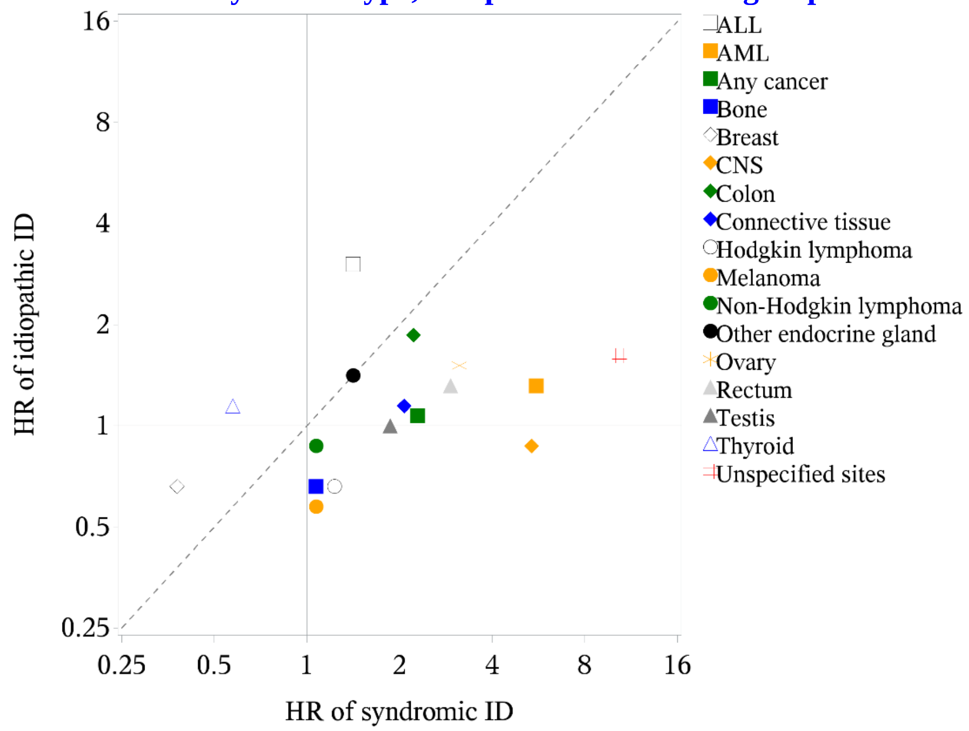

.

*** Reviewer #2:

Overall the manuscript is written clearly and concisely, the topic is relevant, and the research extends existing knowledge. I would recommend this manuscript be published in PLOS medicine with some revisions.

Response:
Thank you for the encouraging comments.

Comment: One of the most important revisions I suggest is to explain further what the hazard ratio statistic means, as this could easily be misunderstood. For example, when you say 'a 1.6-fold increased risk of any cancer' on page 12, tell the reader how they should interpret that.

Spruance SL, Reid JE, Grace M, Samore M. Hazard ratio in clinical trials. Antimicrobial agents and chemotherapy. 2004 Aug;48(8):2787-92.

Sutradhar R, Austin PC. Relative rates not relative risks: addressing a widespread misinterpretation of hazard ratios. Annals of epidemiology. 2018 Jan 1;28(1):54-7.

Response:
Thanks for the comment. We have now added the interpretation of hazard ratio using the citation suggested by the reviewer in Methods (Page 10, Lines 187-188):

"*HR is the instantaneous relative rate of cancer at any time during follow-up among individuals with ID, compared to individuals without ID [40].*"

Comment: More Minor recommendations:
You could change your title to include the fact that you did a population-cohort study in Sweden, this makes the life of future researchers easier when they come across your study.

Response:
We have now adapted the title to "Cancer risk in individuals with intellectual disability: A population-based cohort study".

Comment: P. 5 - The first paragraph with the definition of ID is very clear. However, you could also give examples of the most common forms of ID to give the reader some context.

Response:
We have added an example in Introduction (Page 5, Lines 78-80):

"*For instance, individuals with ID generally have an abnormal level of intelligence quotient (IQ) (under 70) and deficiency in at least two adaptive behaviours in environment and social milieu [6].*"

Comment: P. 6 - Does 'Nordic mothers in Sweden' mean that everyone is of the same ethnicity or with is it simply related to citizenship? Was this something recorded in the data you had? This does not need to be something you add as a covariate into analyses necessarily, but it is interesting to know the diversity of the population you looked at.

Response:

The term “Nordic mothers” used in the original manuscript means mothers born in Nordic countries. This does not directly correspond to ethnicity or citizenship, although vast majority of these mothers are indeed Caucasian. We have now replaced this term with “mothers born in a Nordic country” throughout the manuscript.

Comment: P. 7 - You could define what the Apgar score is and how it is relevant, as not everyone will know this.

Response:
We have now added the definition of Apgar score and its relevance to ID and cancer in Methods (Pages 8-9, Lines 161-164):

“*Apgar score is a common measurement of immediate health status of infants on the basis of skin complexion, heart rate, muscle tone, reflex irritability and respiration effort after delivery. Apgar score has been shown to be associated with cognitive function as well as risk of childhood cancer [36,37].*”

Comment: P 7 - You said you 'obtained information about paternal age at delivery through Multi-Generation Register' - But, I wondered if you were successful at tracing all fathers? If not, did that impact some of your analysed sample sizes?

Response:
In the final analytic cohort, there were 23,229 children (0.65%) with missing information on their biological fathers. Because of this small proportion, the exclusion of children because of this missingness has believably negligible impact on the results. We have clarified this in Methods (Page 7, Lines 121-124):

“*We first identified a total of 3,557,910 individuals in the study cohort* ***(S1 Fig)****. Individuals with no information on father (N=23,229), no information on sex (N=7), or inconsistent information (emigration, death, or cancer diagnosis before birth, N=3,369), were excluded from the analysis. The prespecified analysis plan is presented in the* ***S1 Text****.*”

Comment: P. 9 - I have a few comments around the terms used for sex.

* You include sex as a covariate in some analyses, but you do not state whether the levels for this are 2 (Female, Male) or 3 (Female, Male, Intersex). Please define this as you do for covariate such as severity of ID.
* Also, the terms sex and gender, though commonly used interchangeably in research, are seen as two distinct aspects of an individual's lived experience.
* Best current practice is to refer to sex at birth (Female, Male, Intersex/Undetermined) and gender identity (woman, man, non-binary, indigenous or other cultural gender minority [e.g., two-spirit], etc.,).
* Slade T, Gross DP, Niwa L, McKillop AB, Guptill C. Sex and gender demographic questions: improving methodological quality, inclusivity, and ethical administration. International Journal of Social Research Methodology. 2020 Sep 12:1-2.
* Most relevant to your article is sex at birth of course, but on pages 12 and 18 you refer to 'men and women'. It would be more appropriate for you to refer to male and female if you are interested in sex at birth rather than gender identity

Response:

Thanks for the comment. We now use the terms “sex”, “males” and “females” throughout the manuscript and clarify the level of sex (males or females) in the Methods (Page 10, Lines 190-191):

“*In model 1, analyses were adjusted for sex (male/female) and birth year.*”

Comment: P. 9 - Do you have a citation for 'the risk of both ID and cancer is increased in preterm born individuals,' ?

Response:
We have now added two references for the sentence (Page 11, Lines 214-215):

“***(3)** As the risk of both ID and cancer has been suggested to be increased in preterm born individuals [41,42], we repeated the main analysis among individuals born preterm.*”

Comment: P. 10 - Do you have any code that you could share for your statistical analysis?

Response:
We have now uploaded our SAS codes for data management and main analysis in supplementary file and stated this in Methods (Page 11, Lines 229-230):

“*The SAS codes for data management and main analysis are presented in **S2 Text**.*”

Comment: P. 11 - The result you give for point (1) in your supplementary and sensitivity analyses section is very short and could be a bit clearer. In particular, what do you mean by 'similar'?

Response:
We have now rephased the sentence (Page 13, Lines 260-261):

“*The sibling analysis yielded similar point estimate of risk for any cancer in relation to ID as in the population analysis **(Table 3)**.*”

Comment: P. 12 - Be careful with using the word 'greatly', perhaps remove and just say that it 'did not vary by sex, ID severity' etc., as that is what you found.

Response:
We have removed “greatly” as suggested (e.g., Page 14, Lines 286-288):

“*The risk of any cancer was higher for syndromic ID and childhood cancer, but did not vary by sex, ID severity, birth weight, Apgar score at 1 minute, gestational age, parental education, maternal smoking during pregnancy, or calendar year of birth.*”

Comment: P. 12 - Similarly, you say 'not predominantly due to factor shared by siblings', but did you show that it was related to this at all? This relates back to your explanation of point 1 in sup/sensitivity analysis in results.

Response:
We have now rephased the sentence (Page 14, Lines 288-289):

“The sibling comparison showed no support for familial confounding to the observed association.”

Comment: P. 12 - You mention that there is delay and under-diagnosis of cancer in people with IDs. This is an extremely impactful point that would be worth explaining a little further to the reader.

* Firstly, give a citation for your statement (e.g., Hogg and Tuffrey-Wijne, 2008, and say why is this could be the case (i.e. communication difficulties leading to lack of pain reporting - Hogg and Tuffrey-Wijne, 2008; Millard and de Knegt, 2019).
* Hogg J, Tuffrey-Wijne I. Cancer and intellectual disability: a review of some key contextual issues. Journal of Applied Research in Intellectual Disabilities. 2008 Nov;21(6):509-18.
* Millard SK, de Knegt NC. Cancer pain in people with intellectual disabilities: systematic review and survey of health care professionals. Journal of pain and symptom management. 2019 Dec 1;58(6):1081-99.
* Related to this, do you know anything about the stage of cancer at diagnosis in your data? You may have been able to show that there is delayed diagnosis of cancer in people with IDs.

Response:

Thanks for the great comment. We agree with the reviewer that there might be diagnostic delay or under-diagnosis of cancer among individuals with ID. As we, unfortunately, have no information on cancer stage in the present study, we cannot compare the cancer stage at diagnosis between individuals with and without ID. However, individuals with ID had a higher incidence of cancer diagnosed as “other or unspecified sites” than individuals without ID, indicating that individuals with ID are more likely to have a cancer diagnosis of unknown origin or at metastatic stage.

We have now added comments regarding this in the Discussion including the references suggested by the reviewer (Page 14-15 Lines 296-302):

“Since individuals with ID usually have difficulty in communicating signs and symptoms, they might experience delayed cancer diagnosis or under-diagnosis of cancer [44,45]. Although we had no data on cancer stage in the present study, we did notice a higher incidence of cancer in other or unspecified sites (i.e., cancer of unknown origin or diagnosed at metastatic stage) among individuals with ID, compared with others. The diagnostic delay and under-diagnosis of cancer among individuals with ID could have led to an underestimation of the studied association.”

Comment: P. 13 - You say that previous studies had smaller numbers and shorter durations, it may be useful to mention how large and long these studies where, and also in what populations. This could be done either in your introduction or here in the discussion. Having this clearly means that the reader understands the full scope of the study you've done, and if your study is so much bigger, you should display that.

* On a related point, how long is 'enough follow-up'?
* Additionally, did you use any reporting guidelines?

Response:

We have now added the sample size (number of individuals with ID), length of follow-up, and source of study population in the Discussion (Page 16, Lines 330-334):

"*Patja et al. [28] studied 2,173 individuals with ID in a 30-year follow-up study of a Finnish population whereas Sullivan et al. [29] studied 9,409 individuals with ID in a 19-year follow-up study of a population in Western Australia. With a total number of 27,956 individuals with ID and a follow-up of 43 years, the present study represents therefore the greatest effort of its kind to date*."

We have now added a STROBE checklist as a supplementary file and referred to it in the Methods section (Page 11-12, Lines 231-233):

"*This study is reported according to the Strengthening the Reporting of Observational Studies in Epidemiology (STROBE) guideline (**S3 Text**).*"

*** Reviewer #3:

Please note that this is a statistical review - I cannot comment on the importance of findings, only the validity.

--

This paper assesses whether individuals with intellectual disability are more likely to suffer from cancer than those without intellectual disability (ID) using registry data from Sweden. The authors additionally perform analyses to assess whether the association is likely to relate to ID in a causal sense.

Comment 1. Due to the design (the oldest individuals in the analysis are 43 years old, and the vast majority of years-at-risk are in much younger individuals), this analysis focuses on cancers of young and early-middle age. This should be clearer in the manuscript. For example, the title could state: "Cancer risk in individuals with intellectual disability: analysis of registry data on young and early-middle age Swedish participants" (or similar).

Response:
Thanks for the suggestion. We have now changed the title to "Cancer risk in individuals with intellectual disability: A population-based cohort study" as suggested by the editors.

We have clarified the relatively young age of the study participants in Abstract (Page 2, Line 29-31):

"*All individuals were followed from birth until cancer diagnosis, emigration, death or December 31, 2016 (up to age 43), whichever came first.*"

We have further clarified the relatively young age of the study participants in the manuscript.

In Methods (Page 9, Lines 172-173):
"The individuals were followed from birth until cancer diagnosis, emigration, death or December 31, 2016 (up to age 43), whichever came first."

In Discussion (Pages 14, Lines 282-284):
"In the largest prospective population-based cohort study to date, we observed a 1.6-fold increased risk of any cancer among individuals with ID, compared with individuals without ID, up to age 43."

In Discussion (Page 15, Lines 313-315):
"Despite the large sample size, our study could only address cancer cases diagnosed up to 43 years of age and have limited power to assess the risk of cancers mostly diagnosed at later age, e.g., prostate cancer and lung cancer."

Comment 2. Is the definition of syndromic ID standard? It seems somewhat narrow to my untrained eye.

Response:
There is no perfectly standard definition for syndromic ID in clinical or research setting. The distinction between syndromic ID and idiopathic ID (also named as non-syndromic ID or

"pure" ID) is controversial to date. The motivation to distinguish syndromic ID and idiopathic ID was to raise awareness of the distinctive clinical features, prognosis, and comorbidities in some classical syndromic forms of ID, such as Down syndrome and Fragile X syndrome. With the evolution of the concept, various non-classical dysmorphic features and congenital anomalies, including those in organ systems outside of the central nervous system, are also suggested in a syndromic subclassification.

In today's practice, syndromic ID is defined as a disorder with a clinically defined pattern of somatic abnormalities (e.g., other organ involvement or dysmorphic features) and a phenotype that includes ID. The assumption is that there is a genetic cause in most cases, even if not always identified. Clinically, the distinguishment between syndromic and idiopathic ID usually depends on the presence of congenital malformations or specific features or signs (such a typical gestalt) that indicate underlying congenital malformations or chromosomal abnormalities.

In the present study, based on the Swedish Patient Register, we used information on clinical diagnosis of congenital malformations or chromosomal abnormalities to distinguish syndromic ID from idiopathic ID. We defined syndromic ID as ID with existing congenital malformations or chromosomal abnormalities. We believe that this definition reflects not only the clinical practice but also the principle and original motivation of identification of syndromic ID.

We have clarified this in Methods (Pages 7-8, Lines 139-143):

*"In the study, syndromic ID was defined as individuals with ID and co-existing congenital malformations or chromosomal abnormalities, whereas idiopathic ID was defined as individuals with ID and without co-existing congenital malformations or chromosomal abnormalities. ICD codes for ID, ID severity and ID type are listed in **S1 Table**."*

Comment 3. While I understand that some of the motivation for the investigation comes from the desire to analyse rare cancers, some of these cancers are very rare. Aside from the wide confidence intervals in some analyses, this also leads to an upward bias in estimates that results from only including cancers with at least one case. If a cancer had no cases amongst individuals with ID, then this cancer would not be included in the analysis. The restriction to cancers with at least one case means that for very rare cancers, a positive hazard ratio (HR) is guaranteed. Additionally, as an individual can contribute to more than one cancer, it may be that several of the positive HR estimates are due to a single individual with multiple rare cancers - while the wide confidence intervals indicate imprecise estimates, they aren't able to inform the reader whether these are multiple independent wide estimates, or multiple dependent wide estimates taking positive values due to one or two multimorbid individuals.

For this reason, I would suggest either dropping the analyses of cancers with small numbers of cases in individuals with ID (eg only cancers with >5 cases), or greater aggregation of cancer outcomes, to include cancer types with no cases in individuals with ID - this is important to ensure that estimates are not biased by the exclusion of cancer types with zero cases. Greater aggregation of cancer outcomes would also ensure that individuals with multiple cancer types do not have an undue influence on analyses.

Response:

Thank you for raising this concern. Most previous studies have focused on selected cancer types. The rationale of the present study, to examine the risk of all different cancer types, was to provide a holistic description of cancer risk and the distribution of cancer types among individuals with ID, without any prior selection. We believe this information is relevant, especially for physicians caring for individuals with ID and family members of individuals with ID, and compliments importantly the existing literature.

Further, we do not believe that our inference is greatly (upward) biased. First, we did not observe any statistically significant risk increase for the cancer types where there was only one case among individuals with ID (cancers in the salivary gland, gland, or eye and non-melanoma skin cancer). Second, among individuals with ID, only 1 (0.004 %) had been diagnosed with more than one cancer (primary cancer: endocrine gland cancer; secondary cancer: non-Hodgkin's lymphoma), whereas among individuals without ID, 422 (0.012 %) had more than one cancer diagnosis.

We have added this information in Methods (Page 8, Lines 154-156):

"An individual with more than one cancer type (one individual with ID and 422 individuals without ID) was counted as a cancer case in the analysis of several cancer types."

Regardless, we agree with the reviewer's concern in principle and, as suggested, have performed an additional analysis using larger categories of cancer types (i.e., by organ system, please see the below S11 Table and S3 Table for the new categorization of cancer types). We have added the analysis to Methods and Results and discussed the concern of limited numbers of some cancer types in Discussion.

S11 Table. Incidence rates (IRs, per 100,000 person-years) and hazard ratios (HRs) with 95% confidence intervals (CIs) of cancer among individuals with intellectual disability (ID) by organ systems, compared to reference group

**Cancer categories**	**Model 2[a]**
Buccal cavity and pharynx	0.6 (0.1-4.4)
Digestive system	2.9 (1.8-4.6)
Respiratory system	0.8 (0.1-5.4)
Breast and reproductive system	1.2 (0.8-1.6)
Urinary system	2.8 (1.3-6.3)
CNS[b]	2.7 (2.0-3.7)
Hematological malignancy	1.5 (1.1-2.2)
Other categories	1.2 (0.9-1.6)

[a] Analyses adjusted for birth year (as natural cubic splines), sex, maternal and paternal age at delivery, maternal and paternal psychiatric disorder history at delivery, maternal and paternal cancer history at delivery.
[b] CNS refers to central nervous system.

In Methods (Page 11, Lines 222-225):
"***(8)*** *The analyses of individual cancer types with at least one case among individuals with ID may introduce bias. In a sensitivity analysis, we studied cancer types by larger categories (i.e., by organ systems) regardless of the number of cases among individuals with ID.*"

In Results (Page 13, Lines 275-277):
"***(8) Cancer groups by organ systems****: we observed statistically significantly higher risks for cancers in the digestive system, urinary system, CNS as well as hematological malignancies* ***(S11 Table)****.*"

In Discussion (Page 15, Lines 310-313):
"*Restricting analysis to individual cancer types with at least one case among individuals with ID might have introduced bias to some extent. Interpretation of the cancer-type specific results should therefore be cautious.*"

Comment 4. Page 7: "To adjust for potential confounding or effect modification..." - does covariate adjustment account for effect modification?

Response:
Thanks for pointing out the confusion. We have now rephased the sentence (Page 8, Lines 158-161):

"*We collected information on variables from the Medical Birth Register, including sex, birth year, maternal age at delivery, maternal smoking during pregnancy (at 1st antenatal visit), multiple birth, gestational age (week), birth weight (kg), and Apgar score at 1 minute [31] to be studied as potential confounders or effect modifiers for the studied association.*"

Comment 5. The list of covariates on page 7 is somewhat confusing. My initial reading was that these listed covariates would be adjusted for in the analysis. But on closer reading, these variables are only adjusted for in supplementary analyses? And it's not clear from eTable 8 that there is any analysis performed that adjusted for all of these variables? - you stratify on some of them, but I think you only adjust for at most one of these at a time?

I understand that the main analyses should be careful not to over-adjust (as potentially some of these variables are mediators), so for me, this is a case of clarifying the text rather than changing the analysis.

Response:
We have modified the Covariates section in Methods (Page 8, Lines 158-161) to clarify:

"*We collected information on variables from the Medical Birth Register, including sex, birth year, maternal age at delivery, maternal smoking during pregnancy (at 1st antenatal visit), multiple birth, gestational age (week), birth weight (kg), and Apgar score at 1 minute [31] to be studied as potential confounders or effect modifiers for the studied association.*"

We have also modified the description of the sensitivity analysis to clarify the different adjustment strategies used in different analyses (Pages 10-11, Lines 207-214):

"***(2)** To further explore the impact of birth characteristics, parental education and maternal smoking during pregnancy as potential confounders on the association of ID with cancer, we, in separate models, adjusted for gestational age (<37, 37-41, >41 weeks), birth weight (<2.5, 2.5-4, >4 kg), Apgar score at 1 minute (≥7, 4-6, ≤3), multiple birth (yes or no), maternal and paternal educational level (<9, 9-12, >12 years in school) and maternal smoking during pregnancy (yes or no). We also performed subgroup analyses by these variables to assess whether they could be effect modifiers of the studied association.*"

Comment 6. Page 8: "Attained age was used as the underlying time scale" - what is attained age?

Response:
We have now replaced "attained age" with "age at follow-up" in Methods (Page 10, Lines 188-189):

"*Age at follow-up was used as the underlying time scale.*"

Comment 7. Page 9: "We examined the effect of intelligence quotient (IQ) score on cancer risk through deriving IQ score from the ICD diagnostic codes." - please be careful of causal language.

Response:
We have rephased the sentence (Page 10, Lines 206-207):

"*We derived IQ score from the ICD codes and examined the association between IQ and cancer risk (**S1 Table**).*"

Comment 8. Tables 2-4 may be clearer as figures. It's difficult to take in and compare all the HRs - would be more impactful if the reader could scan a column of visual estimates rather than have to read all the numbers.

Response:
We agree and have now replaced Table 3 with S3 Fig and Table 4 with Fig 1. However, as we believe it is important to provide incidence rates for different cancer types, we decided to keep Table 2 as is. We are certainly willing to reconsider our position if the reviewer would disagree with us.

Comment 9. Would be good to include the headline results from the supplementary and sensitivity analyses in the manuscript (eg the HR for all-cancer from the sibling comparision, the HR for the association between IQ and cancer risk, etc).

Response:
We have now added the headline results as the reviewer suggested (Pages 13-14, Lines 263-280):

"***(1) IQ score:** we found no association between IQ level and cancer risk **(S6 Table). (2) Birth characteristics, parental education and maternal smoking during pregnancy:** the association between ID and any cancer did not change by birth weight, Apgar score at 1 minute, gestational age, multiple birth, parental education at delivery, or maternal smoking during pregnancy **(S7 Table). (3) Preterm birth:** among preterm born individuals, we observed an increased risk of small intestine cancer and ovarian cancer in relation to ID **(S8 Table). (4) Childhood cancer:** there was a higher risk of any childhood cancer in relation to ID **(S9 Table). (5) Age-specific risk increase:** by visual inspection **(S4 Fig),** the risk increases of cancer in esophagus, stomach, small intestine, pancreas and other or unspecific sites appear to be more pronounced among older than younger individuals**. (6) Calendar year of birth:** we did not find difference in risk of any cancer when separately analyzing individuals born 1974 to 1993 and individuals born 1994 to 2013 **(S10 Table). (7) Cancer heritability:** the association between ID and cancer was not related to cancer heritability **(S5 Fig). (8) Cancer groups by organ systems**: we observed statistically significantly higher risks for cancers in the digestive system, urinary system, CNS as well as hematological malignancies **(S11 Table). (9) Lag-time analysis for CNS cancer:** we observed a statistically significantly*

higher risk (HR 1.6, 95% CI 1.0-2.4; P=0.034) of CNS cancer in relation to ID after excluding CNS cancers diagnosed within five years after ID diagnosis."

Comment 10. Page 11: "By visual inspection (eFigure 2), cancer risk appears to increase with age for cancer in the esophagus, stomach, small intestine, pancreas and other or unspecific sites among individuals with ID, compared with the reference group" - it's not clear what comparison you are making here.

Response:
Thanks for the comment. In the analysis, we compared the association between ID and cancer types among younger individuals to that of older individuals, through plotting age-specific risk increase. We have now clarified this in Results (Page 13, Lines 269-272):

"***(5) Age-specific risk increase:*** *by visual inspection* ***(S4 Fig),*** *the risk increases of cancer in esophagus, stomach, small intestine, pancreas and other or unspecific sites appear to be more pronounced among older than younger individuals*."

Comment 11. eTable 7: Two of the odds ratios are over 900 million - something not quite right here.

Response:
The extreme odds ratios were due to that there was no lung cancer or eye cancer case among individuals with moderate, severe, profound and unspecified ID. We realize that this is meaningless and have replaced the extreme odds ratios with "Not estimable" in the supplementary S6 Table.

Comment 12. Discussion: Is reverse causation a possibility, particularly for CNS cancers? Could be that pre-clinical cancer increases risk of ID?

Response:
We have now performed a lag-time analysis for CNS cancers in which CNS cancers diagnosed within the years after diagnosis of ID were excluded. As we still observed a statistically significantly increased risk (HR 1.6, 95% CI 1.0-2.4; P=0.034) of CNS cancers in relation to ID in this analysis, the concern of reverse causality should have been relieved to a large extent. We have now added the additional analysis in Methods and Results and discussed the concern of reverse causality in Discussion.

In Methods (Page 11, Lines 225-228):
"*(9)* *To address the possibility of reverse causation (i.e., ID might be subsequent to cancer such as CNS tumors), we performed an additional analysis in which individuals with a CNS cancer diagnosed within five years after the start of follow-up were excluded.*"

In Results (Pages 13-14, Lines 277-280):
"*(9) Lag-time analysis for CNS cancer:* *we observed a statistically significantly higher risk (HR 1.6, 95% CI 1.0-2.4; P=0.034) of CNS cancer in relation to ID after excluding CNS cancers diagnosed within five years after ID diagnosis.*"

In Discussion (Page 15, Lines 308-310):

"ID might be consequent to cancer such as CNS tumor. The increased risk of CNS cancer among individuals with ID even after excluding CNS cancers diagnosed within five years after ID diagnosis alleviated this concern to a large extent."

Comment 13. Abstract: The claim of "detailed adjustment for potential confounding" is somewhat misplaced (in the text, the claim is phrased as: "adjusted for a rich set of potential confounders"). First, it's not clear why the authors want to adjust for large numbers of confounders - if this analysis is simply performed to identify whether individuals with ID are at increased cancer risk, then adjustment for covariates is unnecessary. Secondly, the main model 1 and 2 do not adjust for large numbers of covariates (again, I don't think this is unreasonable). Thirdly, many key covariates (BMI, smoking status, alcohol status, etc) are not available in the dataset. And finally, it's not clear that all the covariates are confounders. So while I value the potential for addressing confounding in the within-family analyses (and would suggest that eTable 6 may be worth including as a main table/figure), I wouldn't say that comprehensive covariate adjustment is a feature of this work (not should it be).

Response:
Thanks for the comment. We agree and have now deleted "detailed adjustment for potential confounding" and "adjusted for a rich set of potential confounders" from Abstract (Page 2, Lines 31-33):

"We fitted Cox regression models to calculate hazard ratios (HRs) and 95% confidence intervals (CIs) as measures of cancer risk in relation to ID after adjusting for several potential confounders."

In Introduction (Page 6, Lines 109-111):
"We examined the association by ID severity and type (i.e., idiopathic or syndromic), and adjusted for several potential confounders including birth characteristics and performed a sibling-comparison to adjusted for potential familial confounding."

In Discussion (Page 14, Lines 294-295):
"The adjustment for potential confounders and the sibling-comparison further strengthen the validity of our findings."

Finally, we have now used the original eTable 6 as a new Table 3 in the revised manuscript.

Comment 14. Overall, the researchers provide convincing evidence that individuals with ID are at greater risk of cancer both when comparing to the general population, and when restricting to matched comparisons within family units. I am cautious about the validity of some of the detailed comparisons of cancer subtypes, and would encourage either focus on a smaller number of cancer subtypes, or aggregation of related subtypes. However, I leave the final decision here to subject-matter experts.

Response:
Thanks for the positive comment. We have revised our manuscript according to the reviewer's suggestion regarding the use of cancer outcomes. Please refer to the response of the third comment of the reviewer.

*** Reviewer #4:

This is a well-structured and well-written article with well-defined title, clear aim. The important finding of this study is the 1.6-fold increase of cancer rate among patient with ID. Regarding methodology, the process of subject selection was clear, variables were defined and measured appropriately, and study methods seemed to be valid and reliable. But I like to request for further checking of methodology by more experienced one. This study also highlighted some possible mechanisms behind this scene, which made it praiseworthy and helpful for further research. From my side, there is no correction needed.

Response:

Thank you very much for the greatly encouraging comments.

*** Reviewer #5:

Comment. This manuscript by Liu et al is one of the largest longitudinal studies that has been carried out to estimate the risk of cancer among individuals with intellectual disability. The study shows that individuals with ID have an increased risk of cancer and suggest extended surveillance/early intervention in cancer.

The manuscript is well written, addresses the strengths and weakness of the study and compares with previous studies. I recommend this manuscript for publication to advance the understanding in this field.

Response:
Thank you for the positive comments.

Comment 1. Following are some of the points that I would like to get some clarifications on:
1. In one of the previous studies by Sullivan et al. 2004 (PMID: 15801486), males with ID were observed to have a significantly increased risk of leukemia, brain and stomach cancers and a reduced risk of prostate cancer, while leukemia, corpus uteri and colorectal cancers were significantly higher in females. Did the authors in the current manuscript investigate it? Authors mention on Page 11 that 'The increased risk of any cancer did not vary by ID severity or between men and women….". Authors should include in the discussion why they do not see the increased risk of cancer among men and women in comparison to Sullivan et al.

Response:
Thanks for the comment. We have added the comparison, and a potential explanation for the difference between our study and the study by Sullivan et al, as well as the study by Patja et al in Discussion (Page 16, Lines 338-343):

"*Our study showed increased risks for colon and uterus cancers in females, CNS cancer in males, as well as leukemia in both males and females, among individuals with ID, which is in line with one previous study [29]. However, we did not observe statistically significantly increased risk of stomach cancer or decreased risk of prostate cancer among males [29] or increased risk of gallbladder and thyroid cancers in general [28], among individuals with ID. The contrasting results may be partly due to different age ranges of studied participants between different studies.*"

Comment 2. eTable5 shows characteristics of individuals with ID by severity of ID. The percentage of different categories of ID were shown for males. Is there a reason why this percentage were not shown for females? Authors should show the percentage of females with breakdown into different ID groups as they have done for males in this Table to make it complete. Looks like out of 27,956 individuals with ID, about 16,221 (58%) were males and about 11,735 (~42%) of them were females.

Response:
The percentage of females can be calculated using 100 minus the percentage of males. We have now added the percentage for females in the Supplementary S5 Table.

Comment 3. Authors also mention that compared with the reference group, individuals with ID were in general more likely to be male. They should mention in the manuscript the

possible reason behind it. We know there is a higher prevalence of ID in males than in females.

Response:

We have now discussed the potential reason of higher prevalence of ID among males in the Discussion (Page 15-16, Lines 320-324):

"*In line with previous research [46], we observed a higher prevalence of ID among males than females. One potential reason is that males have higher vulnerability of X-chromosome linked syndrome, such as fragile X syndrome, which is related to higher risk of ID [47]. Another reason might be differential brain development due to different androgen exposure between males and females [48].*"
