# Supplementary material for: Cancer risk in individuals with intellectual disability in Sweden: A population-based cohort study
Source: PLoS Med. 2021 Oct 21;18(10):e1003840. doi: 10.1371/journal.pmed.1003840 (PMC8568154; doi:10.1371/journal.pmed.1003840)
Supplement: Attachment — Submitted filename: Response to editors and reviewers.docx [file pmed.1003840.s020.docx]

**Requests from the editors:**

**Comment:** We suggest adding "in Sweden", or similar, to the title.

**Response:**

Thanks for the suggestion. We have now adapted the title to “Cancer risk in individuals with intellectual disability: A population-based cohort study in Sweden”.

**Comment:** At line 36 (abstract), prior to the presentation of the quantitative findings, we suggest adding a sentence or two to provide some additional detail: including the higher rate of ID in males, the length of follow-up, and the numbers of cancers in both groups.

**Response:**

We have now added such information in Abstract as the editors suggested (Page 2, Lines 36-40):

“*Compared with the reference group, individuals with ID were in general more likely to be male.* *The median follow-up time was 8.9 and 23.0 years for individuals with ID and individuals without ID, respectively. A total of 188 cancer cases were identified among individuals with ID (IR, 62 per 1000 person-years) and 24,960 among reference group (IR, 31 per 1000 person-years).*”

**Comment:** At line 38, please make that "the esophagus ...".

**Response:**

We have now rephased the sentence as the editors suggested (Page 2, Lines 42-43):

“*……as well as for several cancer types, including cancers in the esophagus (HR 28.4, 95% CI 6.2-130.6; P<0.001) ……*”

**Comment:** At line 46, please remove "Despite the large sample size and high-quality data ..."; we suggest adapting the text immediately after this point to: "The main study limitations were the limited statistical power for the analysis of specific cancer types, and the potential for underestimation of the studied associations (e.g., due to potential under-detection or delayed diagnosis of cancer among individuals with ID).".

**Response:**

We have now rephased the sentence as the editors suggested (Page 3, Lines 50-53):

“*The main study limitations were the limited statistical power for the analysis of specific cancer types, and the potential for underestimation of the studied associations (e.g., due to potential under-detection or delayed diagnosis of cancer among individuals with ID).*”

**Comment:** At line 51, please adapt the text to the style "In this study, we found that ...".

**Response:**

We have now rephased the sentence as the editors suggested (Page 3, Lines 55-56):

“*In this study, we found that individuals with ID showed an increased risk of any cancer, as well as for several specific cancer types.*”

**Comment:** Please add bullets to the individual points in the Author summary.

**Response:**

We have now added bullets to individual points in the Author summary (Page 4).

Please also expand the Author summary so that each subsection has 2-3 points. For example, in the final subsection an additional point could cite a need for further research on incidence of some specific types of cancer.

**Response:**

Thanks for the suggestion. We have now expanded the Author summary by adding the following points to different subsections.

Under the subsection of “**Why was this study done?**” (Page 4, Lines 61-63):

“*Earlier clinical studies have suggested an increased risk of some specific types of cancer among individuals with genetic syndromes known to be associated with ID, such as Down syndrome, Fragile X syndrome, and tuberous sclerosis complex.*”

Under the subsection of “**What do these findings mean?**” (Page 4, Lines 83-84):

“*Future studies are needed to assess the risk of some specific types of cancer, especially cancers mostly diagnosed at later age.*”

**Comment:** Please remove the word "prospective" at line 282 and any other instances (we view this as a retrospective analysis).

**Response:**

We have now removed the word "prospective" in the manuscript as suggested.

**Comment:** Please use the general style "age 43 years" throughout.

**Response:**

We have now used the term “age 43 years” throughout the manuscript.

**Comment:** Noting reference 7, some punctuation should be removed from the author list (to leave "Linn JG, ..."); in reference 45, authors' initials should be used.

**Response:**

We have now adapted the references as suggested.

Reference 7 (Page 19, Lines 403-404):

“*7.	Linn JG, Chuaqui J, Wilson DR, Arredondo E. The Global Impact of Intellectual Disability and Other Mental Disorders in Children. Int. J. Childbirth Educ. 2019;34:14–17.*”

Reference 45 (Page 21, Lines 516-517):

“*James H, Irene T. Cancer and Intellectual Disability: A Review of Some Key Contextual Issues, J Appl Res Intellect Disabil, 10.1111/j.1468-3148.2008.00422.x, 21, 6. 2008. 509-518.*”

**Comment:** We suggest moving S1 Figure to the main part of the paper.

**Response:**

We have now moved S1 Figure as Figure 1 in the manuscript.

Please relabel the STROBE attachment "S3_STROBE_Checklist" and refer to it by this label in the Methods section.

**Response:**

We have now relabeled the STROBE checklist as "S3_STROBE_Checklist" and refer to it by the updated label in the Methods section (Pages 11-12, Lines 242-244):

“*This study is reported according to the Strengthening the Reporting of Observational Studies in Epidemiology (STROBE) guideline* ***(S3_STROBE_Checklist)***.”

**Comments from the individual reviewers:**

***** Reviewer #1:**

**Comment:** This article is definitely among the largest of its kind in terms of sample size. The current form of the article seems streamlined and easier to read. The amendments made in this article are commendable. I would recommend the article to be published in its current form.

The conception of idea for the research as well as its novel finding will definitely be of important concern in the future. It might have a public health impact in the sense that recommendations about screening for malignancies for cases with intellectual disabilities could be based on these findings.

I would like to congratulate the whole team for their efforts.

Thank you.

**Response:**

Thank you for the very encouraging comments.

***** Reviewer #2:**

**Comment:** I believe this research extends existing knowledge well and is impactful due to its large sample and thorough methodology. The authors address all my points appropriately and I believe this article is now clear and strong.

I will allow comments on changes to the statistics to be made by reviewer 3, but I have a few other minor points outlined below. After these points have been addressed, I am satisfied and happy for this work to be published in PLOS medicine.

**Response:**

Thank you for the encouraging comments.

**Comment:** 1)	P.8 Line 154-6, I feel this sentence could be a little clearer: "An individual with more than one cancer type (one individual with ID and 422 individuals without ID) was counted as a cancer case in the analysis of several cancer types"

a.	It is unclear if this means they were put into a completely separate analysis. A little clarity would help.

**Response:**

Thank you for the suggestion. We have now rephrased the sentence to make it clearer (Page 8, Lines 164-166):

“*An individual with more than one cancer type (one individual with ID and 422 individuals without ID) was counted as a cancer case in the subgroup analysis of each cancer type.*”

**Comment:** 2)	Great to see that the authors were responsive to my comments on sex and gender. One last addition to this point, on P.8 Line 158, perhaps add "(male/female)" after the variable "sex" so that the reader knows that intersex was not included in this variable.

**Response:**

We have now added "(male/female)" as the reviewer suggested (Page 8, Lines 168-171):

“*We collected information on variables from the Medical Birth Register, including sex (male/female), birth year, maternal age at delivery, maternal smoking during pregnancy (at 1st antenatal visit), multiple birth, gestational age (week), birth weight (kg), and Apgar score at 1 minute [31] to be studied as potential confounders or effect modifiers for the studied association*.”

**Comment:** 3)	P.10 Line 198, there seems to be a spelling error: "within-sibship"

**Response:**

We have modified the sentence for the sake of clarity (Page 10, Lines 208-211):

“*To rule out familial confounding, i.e., confounding due to time-invariant factors common to the full siblings, we performed a sibling comparison by fitting conditional Cox regression models to a dataset with differently exposed siblings with the family identifier (mother-id and father-id) as strata*.”

**Comment:** 4)	P13 Line 276, possibly another spelling error: "statistically significantly" should be "statistically significant"

**Response:**

We have modified the sentence to clarify (Page 14, Lines 288-291):

“***(8) Cancer groups by organ systems:*** *we observed statistically significant associations for cancers in the digestive system (HR 2.9, 95% CI 1.8-4.6; P<0.001), urinary system (HR 2.8, 95% CI 1.3-6.3; P=0.01), CNS (HR 2.7, 95% CI 2.0-3.7; P<0.001) as well as hematological malignancies (HR 1.5, 95% CI 1.1-2.2; P=0.02)* ***(S11 Table).***”

**Comment:** 5)	P14 Line 294, the adjustment for potential confounders should be considered in relation to comments from reviewer 3, however, maybe at this point you could explain why finding that confounders did not impact results is important?

**Response:**

Thanks for the comment. We have now expanded the sentence to explain the importance of showing that adjustment for confounding did not impact the results (Pages 14-15, Lines 307-310):

“*The adjustment for potential confounders and the sibling comparison could partly eliminate the concern that the observed association was due to confounding (including familial confounding), which further strengthened the validity of our findings*.”

**Comment:** 6)	P17 Line 348, you abbreviate ALL but not AML, is there a reason for this?

**Response:**

Thanks for the comment. AML is the abbreviation of acute myeloid leukemia or acute myelogenous leukemia. However, in the sentence the reviewer mentioned (“*For instance, Down syndrome, one of the leading causes of ID, is related to presence of somatic mutations of GATA1 gene and JAK2 gene, which are associated with acute megakaryoblastic leukemia and ALL [10,11,50,51]*”), we talked about “acute megakaryoblastic leukemia” (a rare form of acute myeloid leukemia), which cannot be abbreviated as AML.

**Comment:** 7)	Double check gramma in the discussion, I think a few changes have been made that the authors should give another once over to improve readability.

**Response:**

Thanks for the comment. We have now performed a thorough proof-reading of the manuscript again.

***** Reviewer #3:**

*** Reviewer #3:

**Comment:** Thanks to the authors for their comprehensive revision of this work. It is reassuring to know that the bias arising from considering cancers with at least 1 case in individuals with ID is not considerable. It is also reassuring to know that the results for rare cancers are not driven by a single individual (or a small number of individuals) with ID who are massively multimorbid.

**Response:**

Thanks for the positive comments.

I have a few comments:

**Comment:** Paragraph on Supplementary and sensitivity analyses - if there are key summary measures or estimates, I'd consider providing these in the manuscript.

**Response:**

Thanks for the suggestion. We have now added main results of these analyses in the Results section.

In Page 13, Lines 278-281:

“***(3) Preterm birth:*** *among preterm born individuals, we observed an increased risk of small intestine cancer (HR 89.7, 95% CI 5.6-1439.8; P=0.002) and ovarian cancer (HR 10.0, 95% CI 2.3-43.6; P=0.002) in relation to ID* ***(S8 Table)****.* ***(4) Childhood cancer:*** *there was a higher risk of any childhood cancer in relation to ID (HR 2.81, 95% CI 2.18-3.62; P<0.001)* ***(S9 Table)****.*”

In Page 13, Lines 284-287:

“***(6) Calendar year of birth:*** *we did not find difference in risk of any cancer when separately analyzing individuals born 1974 to 1993 (HR 2.90, 95% CI 1.75-4.82; P<0.001) and individuals born 1994 to 2013 (HR 2.74, 95% CI 1.92-3.91; P<0.001)* ***(S10 Table)****.*”

In Page 14, Lines 288-291:

“***(8) Cancer groups by organ systems:*** *we observed statistically significant associations for cancers in the digestive system (HR 2.9, 95% CI 1.8-4.6; P<0.001), urinary system (HR 2.8, 95% CI 1.3-6.3; P=0.01), CNS (HR 2.7, 95% CI 2.0-3.7; P<0.001) as well as hematological malignancies (HR 1.5, 95% CI 1.1-2.2; P=0.02)* ***(S11 Table)***.”

**Comment:** Figure 1: For me, this is hard to read, and it's a shame to lose the uncertainty intervals for the estimates. I would suggest a single vertical axis, and paired estimates plotted with the uncertainty interval horizontally - one colour for syndromic, another colour for idiopathic. Similar to a forest plot, but with equal sized points and no pooled estimate. I think a series of paired points and horizontal lines (with estimates along the right hand side?) would provide a more compelling presentation. From a quick search, this is close to what I had in mind: https://eur01.safelinks.protection.outlook.com/?url=https%3A%2F%2Fplos.figshare.com%2Farticles%2Ffigure%2F_Forest_Plot_of_Unadjusted_and_Adjusted_Hazard_Ratios_for_Any_and_Violent_Convictions_%2F1482255%2F1&amp;data=04%7C01%7Cqianwei.liu%40ki.se%7C8f4f8e19e23d4005847808d98405b883%7Cbff7eef1cf4b4f32be3da1dda043c05d%7C0%7C0%7C637685983362021284%7CUnknown%7CTWFpbGZsb3d8eyJWIjoiMC4wLjAwMDAiLCJQIjoiV2luMzIiLCJBTiI6Ik1haWwiLCJXVCI6Mn0%3D%7C3000&amp;sdata=vyQYYPg0yprVQIrtw8cjK%2FP5CuPcYccDBa%2BFTds7%2BCQ%3D&amp;reserved=0 - although with pairs of points, not triples, and using colour. (Just to clarify, this is a suggestion - the authors should decide the optimal way to present their data in consultation with the editors.)

If the authors do make this change, I'd consider similar changes for Supp Figures 2 and 3.

**Response:**

Thanks for the constructive suggestion. We created new figures (see below). However, we find the new figures busy, and to some extend are missing our initial aim. Instead, our original figures provide straightforward information on qualitative comparison across the range of hazard ratios, simply based on the 45-degree line. For instance, in S2 Fig, readers could easily find that risks of any cancer and most cancer types do not vary by ID severity since these risk estimates are distributed close to the 45-degree line. We therefore decided not to replace our original figures. We are however willing to reconsider our position if the editors would disagree with us.

**Figure A. Intellectual disability (ID) type specific hazard ratios (HRs) with 95% confidence intervals (CIs) of cancer among patients with ID by cancer type, compared to reference group.**

**Figure B. Hazard ratios (HRs) with 95% confidence intervals (CIs) of cancer among individuals with intellectual disability (ID) by sex, compared to reference group**

**Figure C. Intellectual disability (ID) severity specific hazard ratios (HRs) with 95% confidence intervals (CIs) of cancer among individuals with ID by cancer type, compared to reference group**

**Comment:** S2 Table - what are the %s? Are they % of the overall population with this cancer? Or % of all cancers?

**Response:**

The “%” represents the percentage of all cancer cases among individuals with or without ID. We have now clarified this in S2 Table.

**Comment:** S6 Table - the inexact phrasing "effect of IQ score" is still in the heading.

**Response:**

Thanks for the suggestion. We have now changed the title of S6 Table to “Association between IQ score and risk of cancer among individuals with intellectual disability, compared to reference group, by cancer type”.

***** Reviewer #5:**

**Comment:** The manuscript is considerably improved from the first submission. I accept it in its current form.

**Response:**

Thank you for the positive comment.
